# Tetrahydrohyperforin Inhibits the Proteolytic Processing of Amyloid Precursor Protein and Enhances Its Degradation by Atg5-Dependent Autophagy

**DOI:** 10.1371/journal.pone.0136313

**Published:** 2015-08-26

**Authors:** Viviana A. Cavieres, Alexis González, Vanessa C. Muñoz, Claudia P. Yefi, Hianara A. Bustamante, Rafael R. Barraza, Cheril Tapia-Rojas, Carola Otth, María José Barrera, Carlos González, Gonzalo A. Mardones, Nibaldo C. Inestrosa, Patricia V. Burgos

**Affiliations:** 1 Instituto de Fisiología, Facultad de Medicina, Universidad Austral de Chile, Valdivia, Chile; 2 Centro Interdisciplinario de Estudios del Sistema Nervioso (CISNe), Universidad Austral de Chile, Valdivia, Chile; 3 Centro de Envejecimiento y Regeneración (CARE), Pontificia Universidad Católica de Chile, Santiago, Chile; 4 Instituto de Microbiología Clínica, Facultad de Medicina, Universidad Austral de Chile, Valdivia, Chile; 5 Instituto de Ciencias Biomédicas, Facultad de Medicina, Universidad de Chile, Santiago, Chile; 6 Center for Healthy Brain Ageing, School of Psychiatry, Faculty of Medicine, University of New South Wales, Sydney, Australia; 7 Centro UC Síndrome de Down, Pontificia Universidad Católica de Chile, Santiago, Chile; 8 Centro de Excelencia en Biomedicina de Magallanes (CEBIMA), Universidad de Magallanes, Punta Arenas, Chile; Torrey Pines Institute for Molecular Studies, UNITED STATES

## Abstract

Alzheimer's disease (AD) is a neurodegenerative disorder characterized by the accumulation of amyloid-β (Aβ) peptide. We have previously shown that the compound tetrahydrohyperforin (IDN5706) prevents accumulation of Aβ species in an *in vivo* model of AD, however the mechanism that explains this reduction is not well understood. We show herein that IDN5706 decreases the levels of ER degradation enhancer, mannosidase alpha-like 1 (EDEM1), a key chaperone related to endoplasmic-reticulum-associated degradation (ERAD). Moreover, we observed that low levels of EDEM1 correlated with a strong activation of autophagy, suggesting a crosstalk between these two pathways. We observed that IDN5706 perturbs the glycosylation and proteolytic processing of the amyloid precursor protein (APP), resulting in the accumulation of immature APP (iAPP) in the endoplasmic reticulum. To investigate the contribution of autophagy, we tested the effect of IDN5706 in Atg5-depleted cells. We found that depletion of Atg5 enhanced the accumulation of iAPP in response to IDN5706 by slowing down its degradation. Our findings reveal that IDN5706 promotes degradation of iAPP via the activation of Atg5-dependent autophagy, shedding light on the mechanism that may contribute to the reduction of Aβ production *in vivo*.

## Introduction

Alzheimer's disease (AD) is the most common neurodegenerative disorder among older adults, and is characterized by the progressive deterioration of cognitive functions and the cerebral accumulation of both extracellular amyloid plaques and neurofibrillary tangles in hippocampal regions of the brain [1]. Amyloid plaques are highly enriched in aggregated amyloid-β (Aβ) peptide species, derived from the sequential proteolysis of the amyloid precursor protein (APP) by the β-site APP cleaving enzyme 1 (β-secretase) [2] and the γ-secretase complex [3]. Despite significant progress in the understanding of AD, further research is needed to elucidate the mechanisms underlying the accumulation of Aβ peptide that ultimately leads to early-onset AD.

Tetrahydrohyperforin (IDN5706), a semi synthetic derivative of hyperforin, the active molecule in the St John's Wort plant (*Hypericum perforatum*), as well as the natural compound hyperforin itself [4,5], have been shown to prevent neuropathological changes in a mouse model of AD [6,7]. Effects of both compounds include reduction in spatial memory impairments, reduction in tau hyperphosphorylation, and reduction in Aβ oligomer accumulation, as well as an increase in long-term potentiation (LTP) [7]. Moreover, IDN5706 is a potent inhibitor of γ-secretase-mediated cleavage of the amiloydogenic carboxy-terminal fragment-β (CTFβ) [7].

In the present study we investigated whether IDN5706 modulates degradative pathways that control APP and Aβ levels. We observed that treatment with IDN5706 resulted in a decline in EDEM1 levels, a key component of the ERAD pathway, leading to a transient increase of immaturely glycosylated APP (iAPP) in the endoplasmic reticulum (ER). We show that in the presence of IDN5706, iAPP was instead efficiently degraded via autophagy in an Atg5-dependent manner. We observed that IDN5706 treatment increased the number of autophagosomal structures, the levels of LC3-II in different cell types, and autophagic flux. Therefore, we propose that IDN5706 exerts its neuroprotective effects (i.e. reduction of Aβ generation) by acting as a novel modulator of two important intracellular protein degradation systems, namely ERAD and autophagy, which in turn favors quality control of APP and early degradation of APP species via Atg5-dependent autophagy at the ER level.

## Materials and Methods

### Chemical reagents and antibodies

Chloramphenicol, chloroquine (CQ), cycloheximide (CHX), N-[N-(3,5-difluorophenacetyl)-L-alanyl]-S-phenylglycine *t*-butyl ester (DAPT), puromycin dihydrochloride, tunicamycin and a cocktail of protease inhibitors were purchased from Sigma-Aldrich (St. Louis, MO). Tetrahydrohyperforin (IDN5706) was obtained from Indena SpA (Milan, Italy). Tetrahydrophyperforin is a semi synthetic derivative of hyperforin (WO 03/091194 A1; WO 2004/106275 A2). The following mouse monoclonal antibodies were used: clone AC-74 to β-Actin (catalog number #A2228; Sigma-Aldrich); clone 37 to Calnexin (catalog number #610523; BD Transduction Laboratories, San Jose, CA, USA) and clone 40 to Bip (catalog number #610978; BD Transduction Laboratories); purified anti-GFP antibody conjugated to Horseradish Peroxidase (anti-GFP-HRP; catalog number #130-091-833; Macs Miltenyi Biotec, Bergisch Gladbach, Germany). The rat monoclonal antibody clone 3F3A to Gp78 was kindly supplied by I. Nabi (Cell and Developmental Biology, The University of British Columbia, Vancouver, Canada). The rabbit serum to GFP was kindly supplied by R. Hegde (MRC Laboratory of Molecular Biology, Cambridge, UK). In addition, the following polyclonal antibodies were used: rabbit antibody to LC3 (catalog number #L8918; Sigma-Aldrich); rabbit antibody to EDEM1 (catalog number #E8406; Sigma-Aldrich); rabbit antibody to Atg5 (catalog number #2630; Cell Signaling Technology, Danvers, MA, USA); rabbit antibody to the cytosolic C-terminal region of APP (anti-tail; catalog number #51–2700; Invitrogen, Camarillo, CA, USA); rabbit antibody to HA epitope (catalog number #PRB-101P; Covance, Princeton, NJ, USA); rabbit antibody to phospho-serine 2448 of mTOR (catalog number #5536; Cell Signalling Technology), and rabbit antibody to total mTOR (catalog number #2983; Cell Signaling Technology). The HRP-conjugated secondary antibodies were from Jackson ImmunoResearch (West Grove, PA, USA). Alexa Fluor 594-conjugated secondary antibodies were from Life Technologies (Grand Island, NY, USA).

### Cell culture, plasmids and generation of cell lines

MCF-7 human breast adenocarcinoma cells, H4 human neuroglioma cells, normal rat kidney (NRK) epithelial cells, and human fibroblasts, were obtained from the American Type Culture Collection (Manassas, VA, USA). Chinese Hamster Ovary (CHO) cells stably expressing human APP_751_ carrying the V717F mutation, referred to as “7PA2 cell line”, has been described before [8,9]. All cell lines were cultured in Dulbecco's modified Eagle's medium (DMEM), supplemented with 10% heat-inactivated fetal bovine serum (FBS; Life Technologies), and penicillin/streptomycin (Life Technologies), in a 5% CO_2_ atmosphere at 37°C. The generation of H4 cells stably expressing HA-tagged APP-F/P-D/A-EGFP has been previously reported [10]. The HA-tagged p-CMV-SPORT2-EDEM1 construct was provided by Kuzuhiro Nagata and has been previously reported [11]. The pEGFP-LC3 construct was provided by Jennifer Lippincott-Schwartz, and has been previously described [12]. The tandem mRFP-EGFP-tagged LC3 construct has been described before [13]. The generation of NRK and H4 cells stably expressing EGFP-LC3 or mRFP-EGFP-LC3, respectively, were performed by transfection with Lipofectamine 2000 (Life Technologies), according to the manufacturer's instructions. Transfected cells were selected in medium containing 1 mg/ml Geneticin/G418 (Life Technologies). Single cell colonies were picked, and those with comparable expression levels selected for further analysis. Cells stably expressing transfected DNA were maintained in medium containing 100 μg/ml G418. Cells were grown to confluence in 12- or 24-well plates, and treated with drugs for further Western blot analysis. Starvation assays were performed in the presence of Earle's Balanced Salt Solution (EBSS) (catalog number #E2888; Sigma-Aldrich).

### Preparation of shRNA lentiviral particles

We generated H4 cells with stably reduced levels of either EDEM1 or Atg5 by introducing shRNA-containing lentiviral particles. The shRNA sequences were cloned into pLKO.1 vector. An shRNA against luciferase was employed as a control. Lentiviral particles were generated by co-transfection of HEK293 cells with the pLKO.1-shRNA constructs (1 μg), VSV-g (1 μg) and p∆8.9 (1 μg). Transfections were performed with Lipofectamine 2000 (Invitrogen) following the manufacture's instructions. Forty-eight hours post-transfection, media containing lentiviral particles were transferred to H4 cells in a 1:2 dilution in the presence of 8 μg/ml polybreen. After 24 h, cells were selected with 3 μg/ml puromycin. pLKO.1 vectors were generated by The Broad Institute (Boston, MA; http://www.broad.mit.edu/genome_bio/trc/rnai.html). We screened a total of three different shRNA sequences for the depletion of each protein, selecting the most efficient one. For EDEM1 and Atg5 the selected sequences were 5’-CATGCGACAGATTGACCAGAT-3’ and 5’-CCTGAACAGAATCATCCTTAA-3’, respectively.

### RNA isolation, RT-PCR, and cell viability assay

Total RNA extraction from cultured cells left untreated or treated with 125 or 250 μM IDN5706 for 16 h was carried out using the Improm II RNA extraction kit (Promega, Madison, WI, USA). cDNA was generated using 1 μg of total RNA and the First-Strand cDNA synthesis kit (Invitrogen). The PCR exponential phase was determined on 22–30 cycles to allow semiquantitative comparisons of cDNAs obtained by identical reactions with GoTaq Flexi DNA polymerase (Promega). We used the following forward and reverse primers to EDEM1: 5’-TCGGGAATTGCCATGGAAGG-3’ and 5’-TCATGAGGTTTCGGCCTGTG-3’, using GADPH as a housekeeping control, and a protocol similar to what has been previously described [14]. Cell viability was assessed by a colorimetric assay with methyl thiazolyl tetrazolium (MTT). Briefly, H4 cells were seeded at a density of 15,000 cells/well on a 96-well plate, and incubated 24 h at 37°C in DMEM supplemented with 10% FBS. Cells were left untreated or treated with 250 μM IDN5706 for 16 h. The culture medium was removed, and cells were incubated with MTT (0.5 mg/ml) 4 h at 37°C. After three washes with phosphate-buffered saline (PBS, pH 7.4), the insoluble formazan product was dissolved in dimethyl sulfoxide (US Biological, Salem, MA, USA), and optical density at 570 nm was determined on a Varioskan Flash microplate reader (Thermo Scientific, Waltham, MA, USA). All experiments were performed in triplicate, and the relative cell viability was expressed as percentage relative to untreated control cells.

### Preparation of protein extracts and Western blotting

Cells were washed in cold PBS and subjected to lysis at 4°C in lysis buffer (50 mM Tris-HCl pH 7.4, 150 mM NaCl, 1 mM EDTA, 1% Triton X-100) supplemented with a cocktail of protease inhibitors (Sigma-Aldrich). Lysates were cleared by centrifugation at 16,000 x *g* for 20 min, and protein concentration determined with a protein assay solution (Bio-Rad Laboratories, Hercules, CA, USA). Samples with equivalent amount of protein were boiled for 5 min with Laemmli SDS-PAGE sample buffer, and analyzed by SDS-PAGE. Proteins were electroblotted onto nitrocellulose membranes, and membranes were incubated sequentially with primary and secondary antibodies for 1 h at room temperature, or overnight at 4°C. Chemiluminescence protein detection was performed using Pierce Western Blotting Substrate (Thermo Scientific). As an internal loading control, β-actin levels were examined on the same nitrocellulose membranes. Quantification of chemiluminiscence signal was carried out using UN-SCAN-IT software (Silk Scientific Corporation, Orem, UT, USA).

### Quantification of secreted Aβ species

7PA2 cells grown in 12-well plates for 24 h in normal medium, were further cultured in low glucose DMEM (catalog number #31885–023; Life Technologies), without fetal bovine serum, in the absence or presence of 250 μM IDN5706 for 16 h. After treatment, the medium was collected, subjected to centrifugation at 1,000 x *g* to remove cellular debris, and used to detect Aβ peptides. A sample of 100 μl of collected medium was tested using a commercial Sandwich Enzyme-linked Immunosorbent Assay (ELISA) specific to Aβ40 (EZBRAIN40) and A**β**42 (EZBRAIN42) [15], according to the manufacturer's instructions (EMD Millipore Corporation, Billerica, MA).

### Fluorescence microscopy and quantitative analysis

Indirect immunofluorescence staining of fixed, permeabilized cells was carried out as previously described [16]. Images of fixed cells were acquired with an AxioObserver.D1 microscope equipped with a PlanApo 63x oil immersion objective (NA 1.4), and an AxioCam MRm digital camera (Carl Zeiss). For quantification of fluorescent signals, 12-bit images were acquired under identical settings avoiding signal saturation, and corrected for background signal on each image. The corrected fluorescent signal in each cell of each image was used in Image J (version 1.44o; Wayne Rasband, NIH, http://imagej.nih.gov) to determine the total integrated pixel intensity. Analysis of colocalization by fluorescence microscopy was performed as we previously described [17]. For each pairwise comparison we obtained three values, M1, M2, and the Pearson’s correlation coefficient, *r*, according to Manders *et al*. [18]. M1 is defined as the ratio (summed intensities of pixels from channel-A for which the intensity in channel-B is above zero):(total intensity in channel-A), and M2 is the converse ratio. M1 and M2 vary from 0 to 1, corresponding to no colocalization and complete colocalization, respectively. The Pearson’s correlation coefficient, *r*, quantitatively evaluates the level of colocalization, with one indicating complete positive correlation and zero indicating no correlation [18]. All values were obtained with the plug-in JACoP [19], as implemented in Image J. Each pairwise comparison was done on ten sets of images, and the mean and standard deviation (SD) of each parameter was calculated. In the colocalization analyses of autophagic flux using mRFP-EGFP-LC3, the total integrated pixel intensity of the EGFP signal was subtracted from the total integrated pixel intensity of the mRFP signal in each cell of each image to obtain the mRFP-only signal.

### Immunoprecipitation and deglycosylation assays

Immunoprecipitation of HA-tagged APP-EGFP stably expressed in H4 cells was performed as we previously described [20]. Briefly, H4 cells were washed in cold PBS and subjected to lysis at 4°C in lysis buffer. APP was immunoprecipitated from soluble extracts with a rabbit polyclonal antibody anti-GFP, using Protein A-Sepharose (Sigma-Aldrich). Aliquots of immunoprecipitates were incubated in denaturation solution (New England BioLabs, Ipswich, MA, USA) 5 min at 95°C, and treated 1 h at 37°C with different glycosidases (New England BioLabs), according to the supplier's instructions. Briefly, incubations with PNGase F, *O*-glycosydase and/or sialidase were performed in the presence of buffer G7 and 1% Nonidet P-40, and incubations with Endo H were in the presence of buffer G5. The efficiency of deglycosylation was tested by SDS-PAGE and Western blot with anti-GFP-HRP antibody.

### Densitometric quantification and statistical analysis

The amount of immunoblot signal was estimated using Image J software version 1.44o (Wayne Rasband, NIH, http://imagej.nih.gov). For each condition, protein bands were quantified from at least three independent experiments. Data analysis was performed using Microsoft Excel for Mac 2011 (Microsoft Corporation). Results are represented in graphs depicting the mean ± SD. Statistical significance was determined by one-tailed *t*-test or two-tailed, paired *t*-test. *P*-values > 0.05 were regarded as not statistically significant, and *P*-values *≤* 0.05 were regarded as statistically significant. In the figures, *P*-values between 0.01 and 0.05 are indicated with one asterisk, *P*-values between 0.001 and 0.01 are indicated with two asterisks, and *P*-values less than 0.001 are indicated with three asterisks.

## Results

### IDN5706 decreases the levels of glycosylated EDEM1

**Fig 1:**
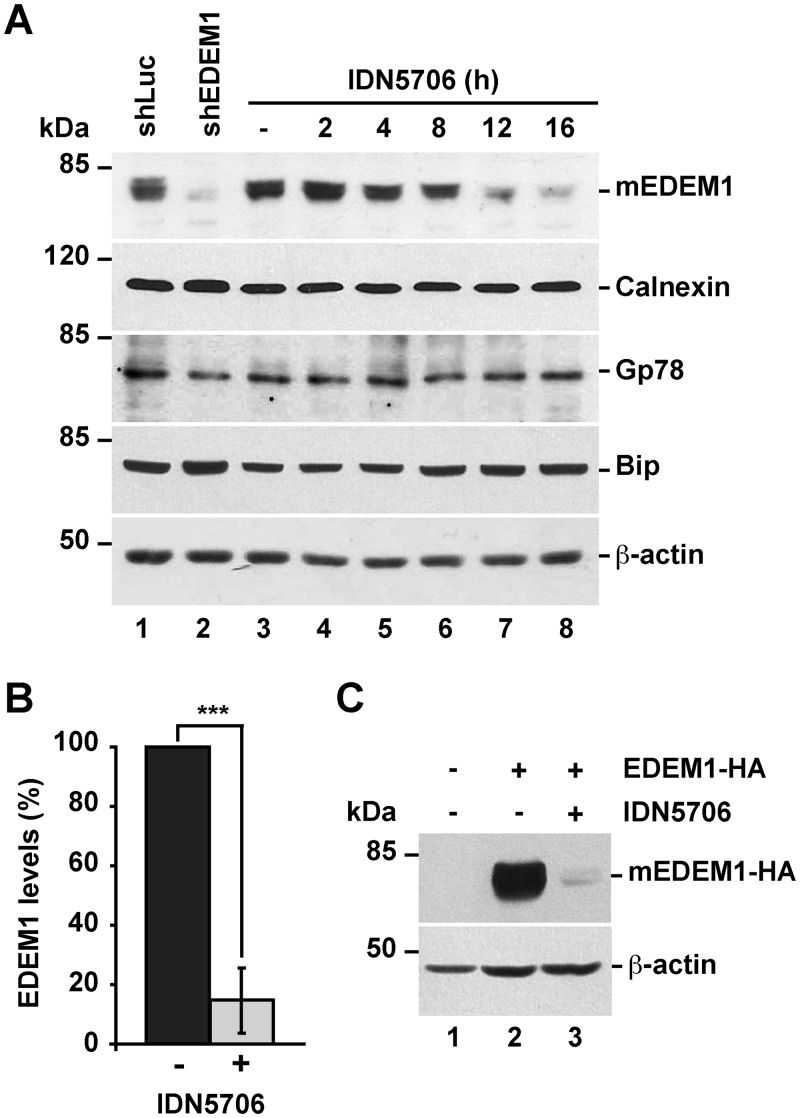
IDN5706 reduces the levels of EDEM1 in a time-dependent manner. (A) H4 cells were left untreated or treated for different periods of time with 250 μM IDN5706, and subsequently analyzed by Western blotting with an EDEM1 antibody. The antibody specificity was validated on protein extracts from cells stably expressing either luciferase shRNA, used as positive control (shLuc), or EDEM1 shRNA (shEDEM1). Specific antibodies against ER proteins including Calnexin, Gp78 and Bip were tested. (B) Densitometric quantification of the levels of EDEM1 in cells left untreated or treated with IDN5706 for 16 h, as shown in A. Bars represent the mean ± SD of three independent experiments (***, *P* < 0.001). (C) H4 cells were either left untreated (lane 1) or transfected with a plasmid encoding HA-epitope-tagged EDEM1 (lanes 2 and 3). After 16 h cells were left untreated or treated with 250 μM IDN5706 for 8 h, and cellular extracts were subjected to SDS-PAGE followed by immunoblot with mouse antibody anti-HA-epitope. In A and C, Western blotting with antibody to β-actin was used as loading control. The position of molecular mass markers is indicated on the left.

Misfolded glycoproteins that fail to attain their correct conformation in the ER are recruited by EDEM1 from the Calnexin/Calreticulin folding cycle, and are retro-translocated for cytosolic degradation via ER-associated degradation (ERAD) [21,22]. Consequently, several studies have shown that EDEM1 levels are crucial for ERAD function [11,21,23,24]. Given that the levels of APP are controlled by ERAD [10,25], we tested whether IDN5706 could modulate EDEM1 levels. First, we confirmed the specificity of the antibody used in this study against EDEM1 comparing H4 human neuroglioma cells stably expressing either luciferase shRNA (used as a negative control; Fig 1A, lane 1), or EDEM1 shRNA (Fig 1A, lane 2). As expected, we observed absence of immunoreactivity only in cells expressing EDEM1 shRNA. Surprisingly, the Western blot analysis of H4 cells treated with 250 μM IDN5706 for 16 h showed a dramatic decrease in EDEM1 in a time-dependent manner (Fig 1A, lanes 3–8), to 14.7 ± 10.1% compared to the levels found in untreated cells (Fig 1B). As a control, we evaluated other ER proteins including Calnexin, Gp78, and Bip, observing that IDN5706 treatment had no significant effect on their levels (Fig 1A). To determine whether IDN5706 exerts its effect during transcription, we next evaluated the expression of EDEM1 mRNA by RT-PCR. We observed that treatment with 125 or 250 μM IDN5706 for 16 h had no effect on EDEM1 mRNA levels (S1 Fig). To evaluate the possible effect of IDN5706 during translation, we next examined H4 neuroglioma cells overexpressing HA-epitope-tagged EDEM1. After 16-h of transfection, cells were left untreated or treated with 250 μM IDN5706, observing a strong reduction in EDEM1-HA levels (Fig 1C). Altogether, these results suggest the possibility that low levels of EDEM1 in the presence of IDN5706 are the result of an increase in EDEM1 turnover.

Previous studies have shown that EDEM1 is expressed as both an unglycosylated form (iEDEM1), and as a more abundant, highly glycosylated form (mEDEM1) [26]. Treatment of H4 cells with tunicamycin for 16 h, to prevent *N*-glycosylation, confirmed that at steady state the majority of EDEM1 is glycosylated (S2 Fig). Because we did not observe accumulation of iEDEM1 in cells treated with IDN5706 (S2 Fig), as we observed with tunicamycin treatment, we concluded that IDN5706 decreases the levels of mEDEM1. We also evaluated whether IDN5706 affects cell viability and found no significant effect compared to untreated cells (S3 Fig). This rules out the possibility that the impact of IDN5706 on EDEM1 levels was simply due to changes in cell viability.

### IDN5706 activates autophagy

**Fig 2:**
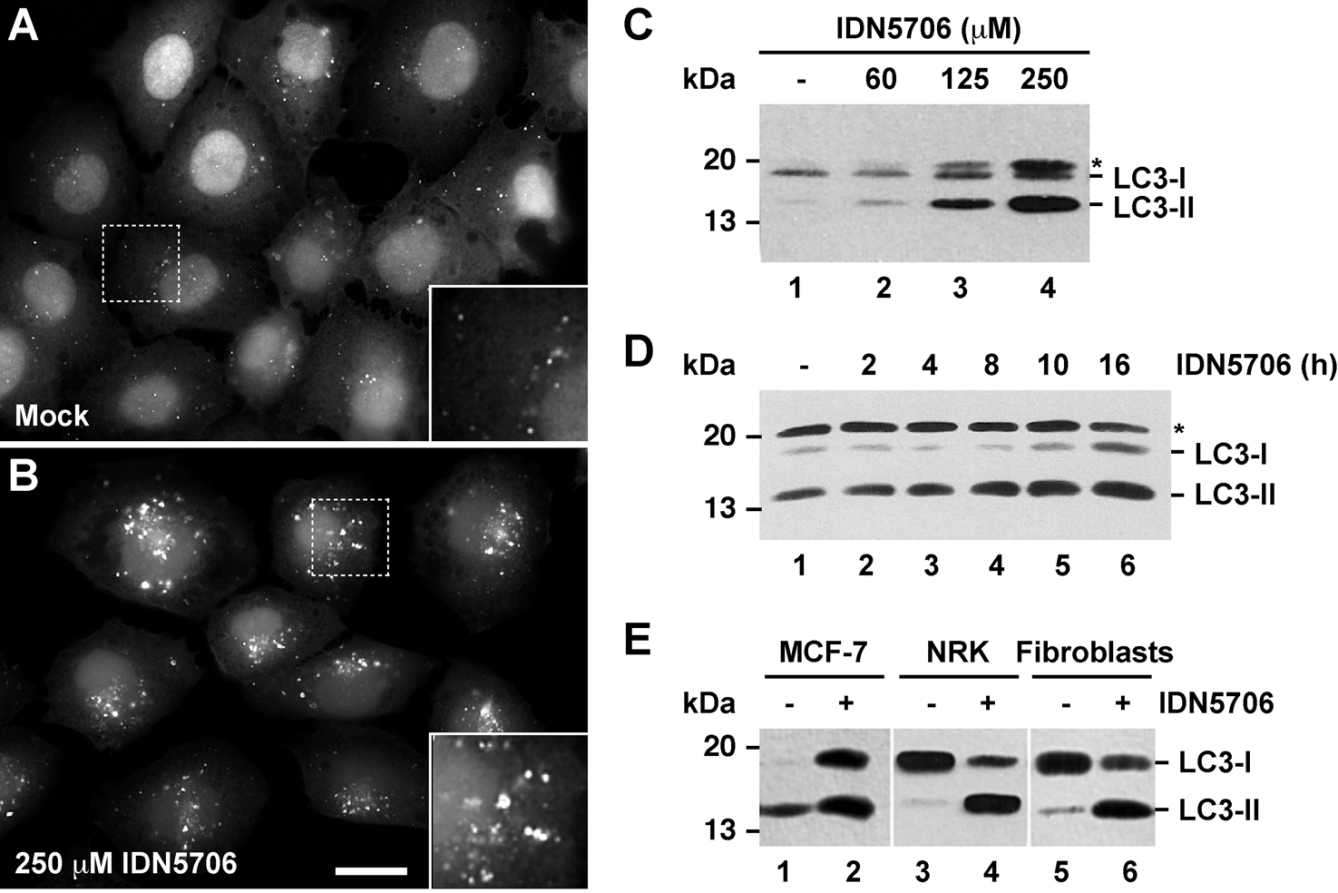
IDN5706 increases LC3-II levels in different cell types. Normal rat kidney (NRK) cells stably expressing GFP-LC3 were left untreated (A) or treated with 250 μM IDN5706 for 16 h (B) and analyzed by fluorescence microscopy. Bar, 10 μm. (C) H4 cells were treated with the indicated concentration of IDN5706 for 16 h. (D) H4 cells were treated with 250 μM IDN5706 for the indicated periods of time. (E) The indicated cell lines were left untreated or treated with 250 μM IDN5706 for 16 h. (C-E) Cell extracts were subjected to Western blot analysis with an antibody to LC3. LC3-I, non-lipidated LC3; LC3-II, lipidated LC3. The asterisk indicates a band detected only in H4 cells (C and D). The position of a molecular mass marker is indicated on the left.

Because disturbances at the ER promote autophagosome biogenesis, either by the accumulation of misfolded glycosylated proteins [27–31], by the impairment of proteasomal activity [32], or by negative regulation of ERAD functionality [33], we asked whether IDN5706 could modulate autophagy. To test this possibility, NRK cells stably expressing EGFP-LC3 were treated with IDN5706 for 16 h and analyzed by fluorescence microscopy. We observed that, compared to untreated cells, IDN5706 increases the fluorescence associated to EGFP-LC3-decorated structures (Fig 2A and 2B). Quantification analysis showed a highly significant, ~20 fold increase in the fluorescent signal of EGFP-LC3 (S4 Fig), a feature indicative of autophagy activation [34,35]. Another hallmark of autophagy is the lipidation of cytosolic LC3-I to LC3-II, which is then incorporated into autophagosome membranes [34,35]. Western blot analysis showed that treatment of H4 cells with IDN5706 increased LC3-II levels in a dose- (Fig 2C) and time- (Fig 2D) dependent manner. We also observed this increase in LC3-II levels in other cell lines (Fig 2E), discarding that this effect is cell-type dependent, and suggesting that IDN5706 is a bona fide activator of autophagy.

**Fig 3:**
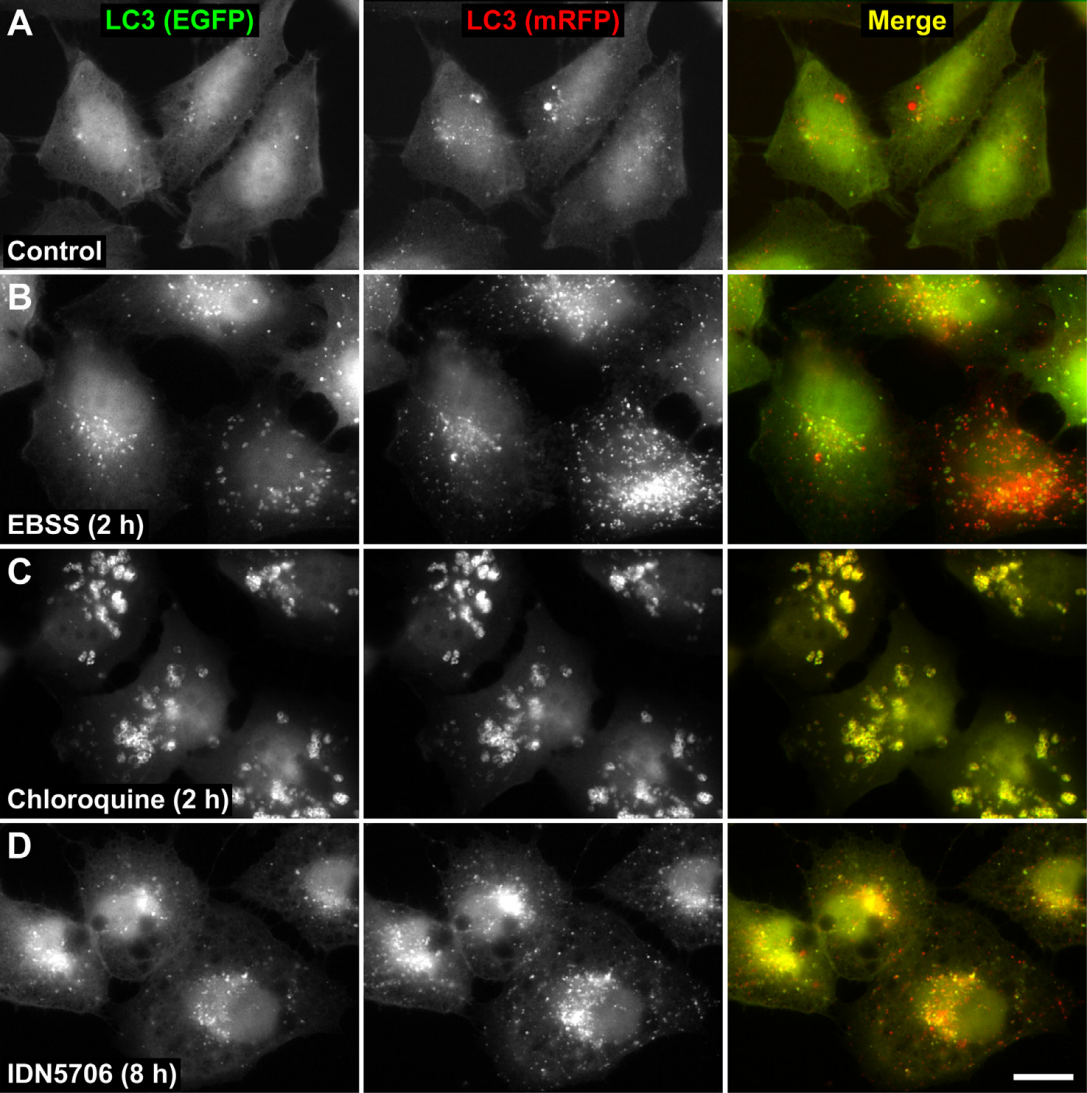
IDN5706 activates autophagy. H4 cells stably expressing mRFP-EGFP-LC3 were left untreated (A) or treated with either EBSS (B), 0.1 mM Chloroquine (C) or 250 μM IDN5706 (D) for the indicated time, and analyzed by fluorescence microscopy. Merging of the images in the green and red channels generated the third image in each row; in merged images red indicates presence of LC3 in acidic compartments (mRFP^+^EGFP^-^), and yellow indicates presence of LC3 in non-acidic compartments (mRFP^+^EGFP^+^). Bar, 10 μm.

We analyzed LC3-II turnover to assess the possibility that increased LC3-II levels upon IDN5706 treatment could be the result of reduced turnover. Cells were pre-treated with IDN5706 for 16 h and, to prevent new protein synthesis, were further incubated for different periods of time with cycloheximide (CHX) and chloramphenicol (CHX-chase) in the presence of IDN5706 (S5 Fig). We observed efficient turnover of LC3-II within the time frame tested, indicating that high levels of LC3-II could be the result of autophagy activation. To evaluate whether CHX treatment could result in an underestimation of the efficiency of LC3-II turnover in the presence of IDN5706 [36], we monitored autophagic flux using mRFP-EGFP-tagged LC3, a convenient assay based on different pH sensitivity of mRFP and EGFP fluorescent proteins [13]. In this assay, while EGFP fluorescence is attenuated by acidic pH, mRFP remains stable. To better characterize this assay in our cell model, H4 cells stably expressing mRFP-EGFP-tagged LC3 were treated with EBSS, a well-known starvation solution used to activate autophagy [37,38]. As expected, we observed that EBSS increased the number of LC3-decorated structures (Fig 3A and 3B). Quantification analysis showed that untreated cells and cells treated with EBBS contained 65.7 ± 3.6% and 70.7 ± 8.0%, respectively, of puncta that were red-only (i.e., mRFP^+^EGFP^-^) (S6 Fig), indicating normal autophagic flux. In contrast, cells treated with Chloroquine (CQ) (Fig 3C), a compound that perturbs fusion between autophagosomes and lysosomes [39,40], showed that only 4.8 ± 0.7% of the LC3-decorated structures were red-only (S6 Fig), an indicative of autophagic flux inhibition [41]. Importantly, when cells were treated with 250 μM IDN5706 for 8 h, a 66.5 ± 5.5% of the LC3-decorated structures showed red-only puncta (S6 Fig), confirming that the increase in LC3-decorated structures in response to IDN5706 treatment is not consequence of autophagic flux inhibition. Taken together, these results demonstrate that IDN5706 activates autophagy.

### Autophagy activation by IDN5706 is dependent on mTOR activity and Atg5 levels

**Fig 4:**
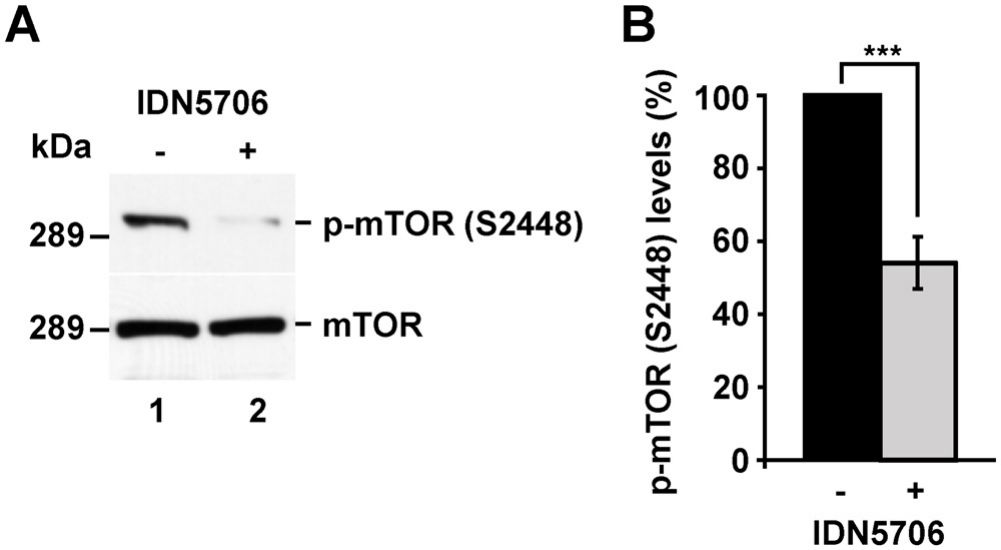
IDN5706 reduces phosphorylation in S2448 of mTOR. (A) N2a cells were left untreated (lane 1) or treated 4 h with 250 μM IDN5706 (lane 2), and cellular extracts were subjected to SDS-PAGE followed by immunoblot with rabbit antibody to phosphorylated mTOR at serine 2448 (p-mTOR (S2448)), or rabbit antibody to total mTOR. Western blotting with antibody to β-actin was used as loading control. The position of a molecular mass marker is indicated on the left. (B) Densitometric analysis of the levels of p-mTOR (S2448) shown in (A). Values were normalized to the levels of total mTOR, and presented as the mean ± SD of three independent experiments. ***, *P* < 0.001.

A key signaling-player in autophagy activation is the phosphorylation state of the protein kinase mammalian target of rapamycin (mTOR) [42]. Lack of phosphorylation at serine-2448 (pS2448) of mTOR is known to activate autophagy, a modification that is used to monitor this pathway [34]. Western blot analysis with an antibody specific to pS2448 shows that treatment of N2a cells with 250 μM IDN5706 for 4 h resulted in a dramatic decrease in the levels of phosphorylated mTOR (Fig 4A), to 54.0 ± 7.1% compared to the levels in untreated cells (Fig 4B), an effect that is persistent with longer treatments (data not shown). This finding suggests that IDN5706 acts as a positive modulator of autophagy through mTOR inactivation.

**Fig 5:**
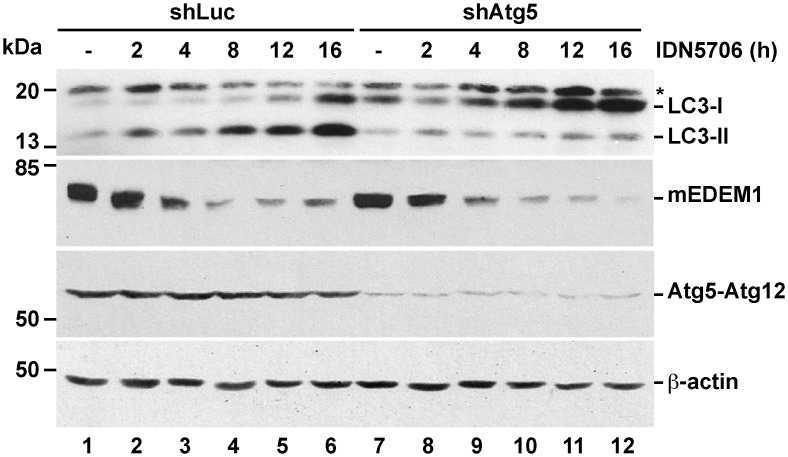
Depletion of Atg5 inhibits IDN5706-induced autophagy, but not EDEM1 degradation. H4 cells stably expressing either luciferase shRNA (control; shLuc) or Atg5 shRNA (shAtg5) were treated with 250 μM IDN5706 for different periods of time. Cellular extracts were subjected to SDS-PAGE followed by Western blot with antibody to LC3, EDEM1 or Atg5-Atg12 complex. LC3-I, non-lipidated LC3; LC3-II, lipidated LC3; mEDEM1, mature EDEM1. The asterisk indicates a band detected only in H4 cells. Western blotting with antibody to β-actin was used as loading control. The position of molecular mass markers is indicated on the left.

Next, we analyzed whether the activation of LC3 lipidation induced by IDN5706 was dependent on Atg5, a protein that forms a conjugate with Atg12 controlling a key step in autophagosome formation [43]. H4 cells stably expressing either luciferase shRNA or Atg5 shRNA were treated with 250 μM IDN5706 for different periods of time (Fig 5). We observed that the generation of LC3-II, induced by IDN5706, was strongly abolished in the absence of Atg5 (Fig 5, lanes 1–6 compared to lanes 7–12). Surprisingly, we found no changes in the levels of EDEM1 upon depletion of Atg5 (Fig 5, lane 1 compared to lane 7). Moreover, we observed that the reduction in EDEM1 levels by IDN5706 was similar in cells depleted of Atg5 (Fig 5, lanes 1–6 compared to lanes 7–12). Altogether, our results show that in H4 cells treated with IDN5706, Atg5 does not control the levels of EDEM1. We conclude that the reduction in EDEM1 levels by IDN5706 occurs by an Atg5-independent turnover pathway. On the other hand, the decrease in LC3-II levels in the absence of Atg5 correlated well with a reduction in autophagic cytoplasmic structures revealed by fluorescence microscopy with an antibody against LC3 (data not shown), confirming that IDN5706 acts as a positive modulator of Atg5-dependent autophagy.

### IDN5706 accumulates endogenous APP, inhibiting its proteolytic processing to carboxy-terminal fragments and Aβ generation

**Fig 6:**
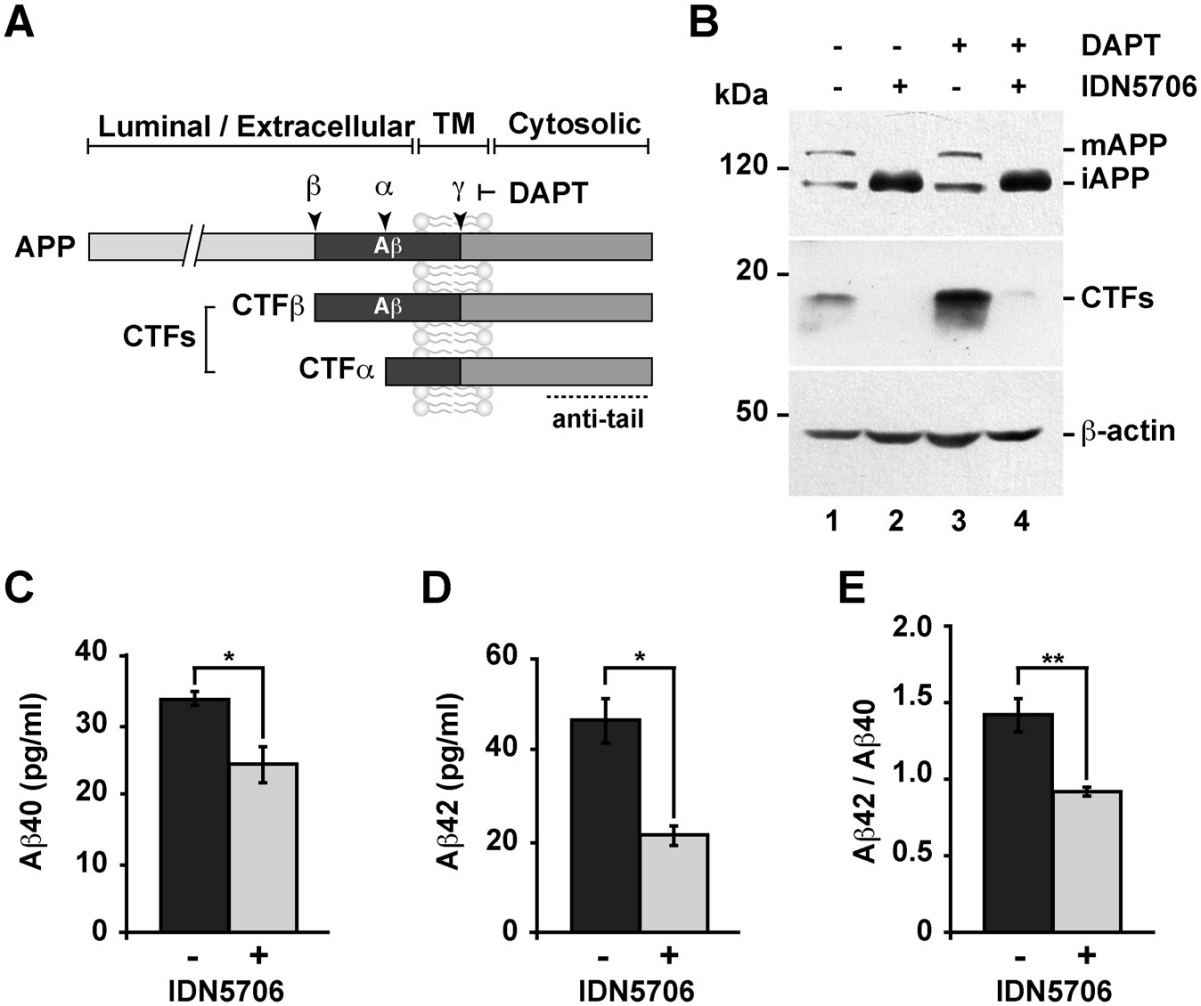
IDN5706 inhibits proteolytic processing of APP to CTFs, and production of Aβ species. (A) Schematic representation of APP and carboxy-terminal fragments (CTFs) indicating their topological domains and the position of the proteolytic cleavage sites by α, β and γ secretases. The cytosolic region, recognized by the anti-tail antibody, and the γ-secretase inhibitor, DAPT, are indicated. (B) H4 cells were left untreated (lane 1) or treated for 16 h either with 250 μM IDN5706 (lane 2), 1 μM DAPT (lane 3), or with a combination of 250 μM IDN5706 and 1 μM DAPT (lane 4). Cell extracts were subjected to Western blot analysis using the anti-tail antibody to the cytosolic C-terminal region of APP. Western blotting with antibody to β-actin was used as loading control. mAPP, mature APP; iAPP, immature APP. The position of molecular mass markers is indicated on the left. (C-D) CHO 7AP2 cells were cultured in DMEM containing low glucose and without fetal bovine serum, in the absence or presence of 250 μM IDN5706 for 16 h. The amount of Aβ40 and Aβ42 peptides in the culture medium was analyzed by ELISA. (E) Ratio of the amount of Aβ42 and Aβ40 peptides as an indicator of toxicity. (C-E) Values are presented as the mean ± SD of three independent experiments. *, *P* < 0,05 and **, *P* < 0,005.

To investigate the possible effects of IDN5706 on APP levels, we used the neuroglioma cell line H4 that we have previously used to investigate APP proteolytic processing [10,20,44]. APP is a type-I transmembrane glycoprotein with a large N-terminal extracellular domain, a single membrane span, and a short C-terminal cytosolic tail (Fig 6A). After synthesis, APP undergoes proteolytic processing by two alternative enzymes called α- and β-secretase that form either of two carboxy-terminal fragments referred as CTFs [45]. A third enzyme called γ-secretase cleaves both CTFs, and cleavage by β- and γ-secretase leads to the generation of the Aβ peptide [45]. Western blot analysis of endogenous APP showed that treatment with IDN5706 produced the loss of mature APP (mAPP) and a strong accumulation of immature APP (iAPP) (Fig 6B), which has a faster electrophoretic mobility compared to *N*- and *O*-glycosylated, mAPP [46]. We also observed that the accumulation of iAPP was time dependent (S7 Fig, lanes 1–6). Next, we studied whether IDN5706 affected the proteolytic processing of endogenous APP, and we evaluated this by using N-[N-(3,5-difluorophenacetyl)-L-alanyl]-S-phenylglycine *t*-butyl ester (DAPT). DAPT is a specific and potent γ-secretase inhibitor that allows the detection of otherwise rapidly processed CTFs. In the absence of DAPT, we observed that accumulation of iAPP with IDN5706 correlated with a marked reduction of CTFs (Fig 6B, lane 2 compared to lane 1). As expected, incubation with DAPT alone caused a steady state accumulation of CTFs (Fig 6B, lane 3 compared to lane 1), consistent with CTFs subsequently cleaved by γ-secretase. However, incubation with DAPT did not preclude the significant reduction in CTF levels in cells treated with IDN5706 (Fig 6B, lane 4 compared to lane 3). Altogether, these results indicate that the treatment with IDN5706 produces the accumulation of iAPP and the impairment of its proteolytic processing. Next, we investigated the effect of IDN5706 treatment on the secretion of Aβ species. We examined the effect of IDN5706 in CHO cells stably expressing human APP_751_ carrying the V717F mutation, a cell line currently referred to as 7AP2, which is extensively used in the study of Aβ secretion [8,9]. We observed a significant decrease in the levels of both, Aβ40 (Fig 6C) and Aβ42 (Fig 6D), to 73.2 ± 7.6% and 45.4 ± 3.2%, respectively, compared to untreated cells. Moreover, we observed a reduction in the Aβ42/Aβ40 ratio to 62.4 ± 0.1%, compared to control cells (Fig 6E). Taken together, these findings demonstrate that IDN5706 precludes the proteolytic processing of APP to CTFs, leading to a significant reduction in the two major species of A**β**.

### IDN5706 disrupts APP glycosylation

**Fig 7:**
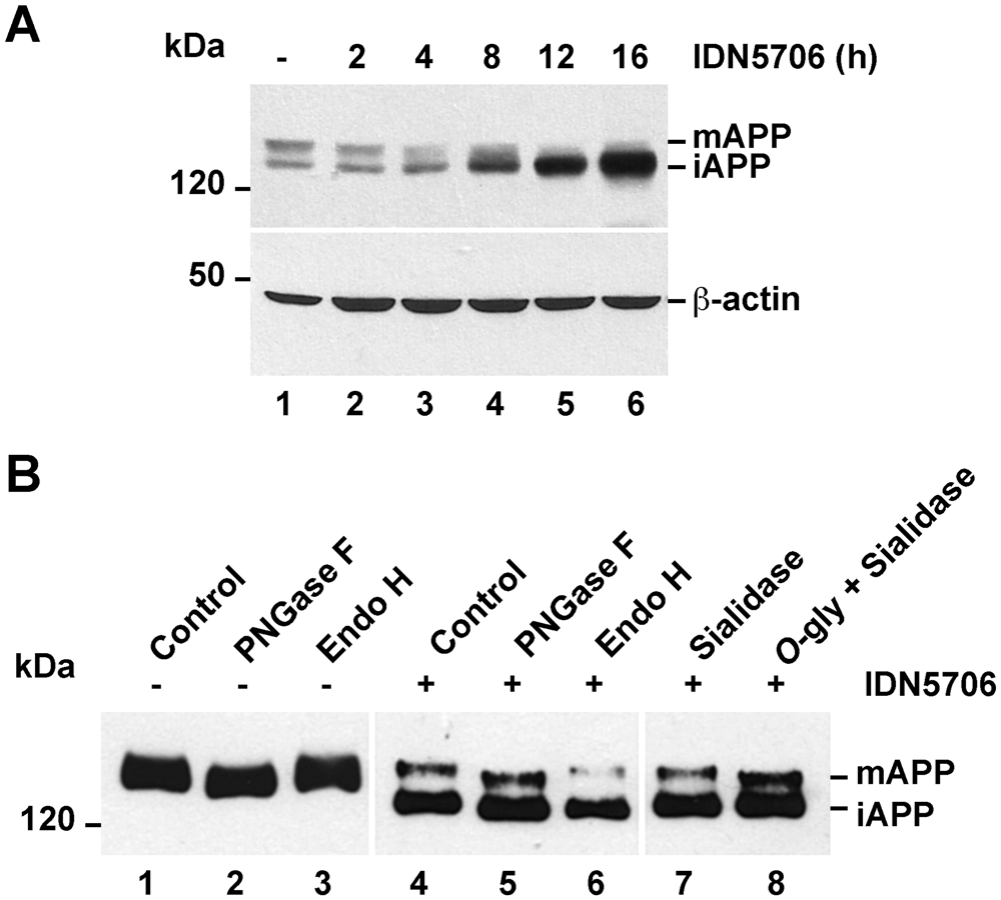
IDN5706 disrupts glycosylation of APP. (A) H4 cells stably expressing an amyloidogenic version of APP tagged to GFP (APP-GFP) were treated with 250 μM IDN5706 for the indicated periods of time. Cell extracts were subjected to Western blot analysis with an antibody to GFP. Western blotting with antibody to β-actin was used as loading control. (B) H4 cells stably expressing APP-GFP, were left untreated (lanes 1–3) or treated for 16 h with 250 μM IDN5706 (lanes 4–8). Cell extracts were subjected to immunoprecipitation with a an antibody to GFP, followed by denaturation and digestion with the indicated glycosidases for 1 h at 37°C. Immunoprecipitated proteins were subjected to Western blot analysis with anti-GFP-HRP. (A-B) The position of molecular mass markers is indicated on the left. mAPP, mature APP; iAPP, immature APP.

Because several studies have shown that autophagy functions as an early quality control mechanism to alleviate abnormal accumulation of immature glycoproteins at the ER [28–33], we evaluated whether accumulated iAPP by IDN5706 treatment in fact corresponded to immaturely glycosylated APP. To investigate this, we stably expressed in H4 cells an amyloidogenic version of APP tagged with green fluorescent protein (APP-GFP), cell line that we have previously described [10]. Western blot analysis showed that IDN5706 treatment produced the accumulation of iAPP-GFP in a time-dependent manner, similar to the effect on endogenous APP (Fig 7A, lanes 1–6). To study whether IDN5706 could disrupt APP glycosylation, cells expressing APP-GFP were treated with IDN5706 for 16 h and soluble extracts were subjected to immunoprecipitation with a polyclonal anti-GFP antibody to capture all APP species. Aliquots of immunoprecipitates were incubated with different glycosidases, followed by Western blot analysis with anti-GFP-HRP (Fig 7B). Digestion with PNGase F, which hydrolyzes nearly all types of *N*-glycan chains from glycoproteins, showed that both forms of APP, i.e., mAPP from untreated cells and mAPP and iAPP from IDN5706-treated cells, are *N*-linked glycosylated (Fig 7B, lanes 2 and 5). To better distinguish between mature and immature *N*-linked glycosylated APP species, we tested sensitivity to Endoglycosidase H (Endo H). Endo H cleaves *N*-linked, mannose-rich oligosaccharides in immature glycoproteins that are en-route in the early secretory pathway between the ER and the first cisternae of the Golgi apparatus. Conversely, Endo H does not cleave highly processed complex oligosaccharides, sugar modifications that occur in glycoproteins at late cisternae of the Golgi apparatus. For this reason, this assay is also used to monitor the extent of glycoproteins localization at the Golgi apparatus after transport from the ER [47]. Incubation with this enzyme resulted in mAPP largely unaffected, both in extracts from cells untreated or treated with IDN5706 (Fig 7B, lanes 3 and 6). In contrast, a drop in the molecular mass of iAPP was apparent in extracts from cells treated with IDN5706 (Fig 7B, lane 6). These findings, along with the PNGase F results, confirm that APP undergoes modification with complex type *N*-linked glycans [46], and strongly indicate that IDN5706 disrupts completion of APP glycosylation. It has been reported that APP is a substrate for additional modifications such as *O*-glycosylation [48]. To investigate whether IDN5706 could affect *O*-glycosylation on APP, we performed deglycosylation using *O*-glycosidase and sialidase. *O*-glycosidase removes *O*-linked galactose-*N*-acetylgalactosamine disaccharides from glycoproteins after cleavage of terminal sialic acids by sialidase [49]. As expected, incubation with sialidase produced a shift in the electrophoretic mobility of mAPP only when coincubated with *O*-glycosidase (Fig 7B, lanes 7 and 8, mAPP). In contrast, iAPP accumulated upon IDN5706 treatment seemed unaffected after incubation with these glycosidases (Fig 7B, lanes 7 and 8, iAPP), indicating absence of *O*-glycosylation. Incubation with *O*-glycosidase alone did not modify mAPP (data not shown), consistent with the presence of terminal sialic acid in *O*-linked glycans. Altogether, these findings indicate that IDN5706 disrupts APP glycosylation, presumably by affecting its intracellular localization.

### IDN5706 accumulates APP at the ER enhancing its degradation by Atg5-dependent autophagy

**Fig 8:**
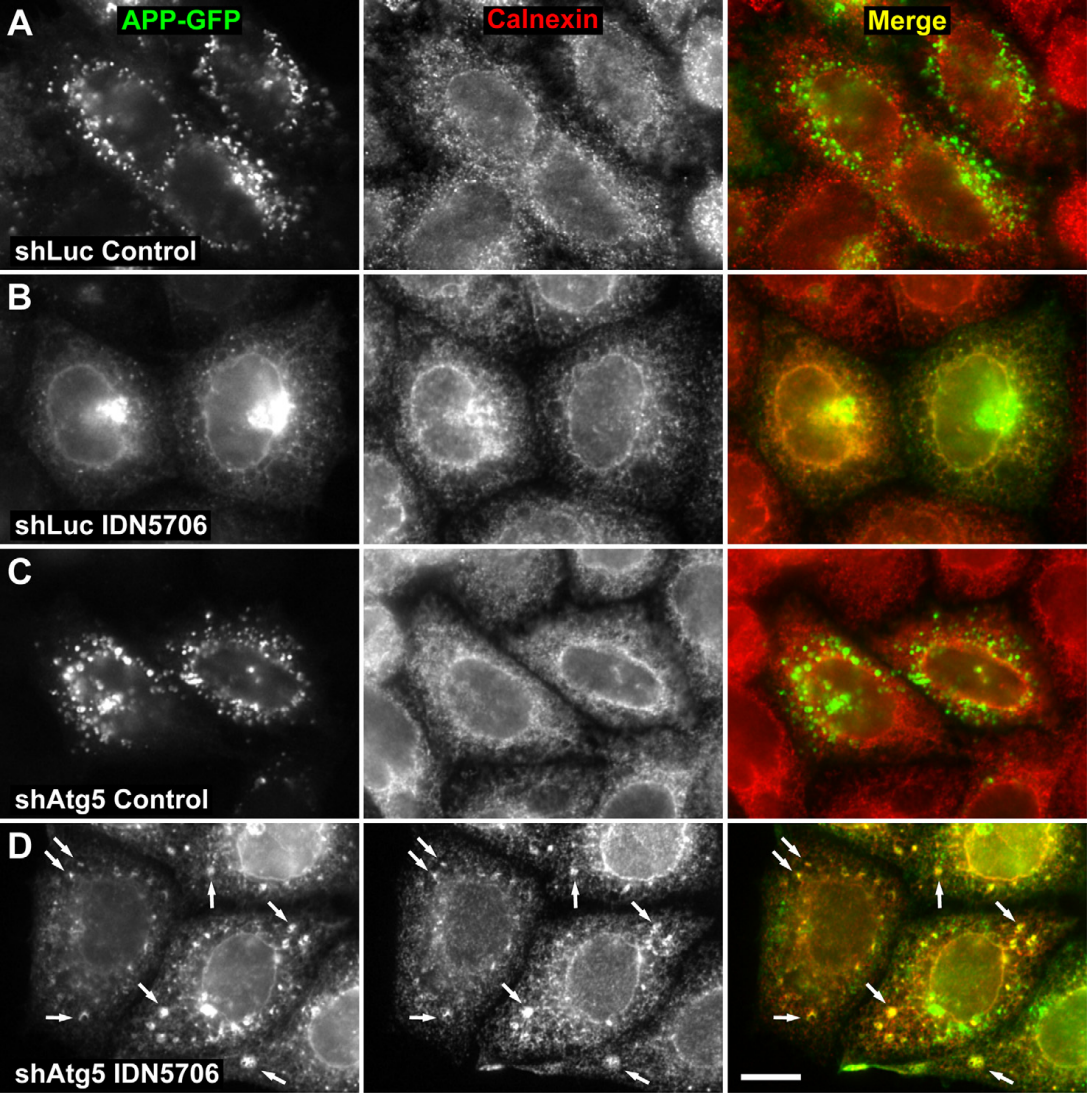
Depletion of Atg5 increases the accumulation of APP in response to IDN5706. H4 cells stably expressing an amyloidogenic version of APP tagged to GFP, and stably expressing either luciferase shRNA (control; shLuc) (A and B) or Atg5 shRNA (shAtg5) (C and D), were left untreated (Control; A and C) or treated with 250 μM IDN5706 for 8 h (IDN5706; B and D). Cells were fixed, and labeled with a mouse monoclonal antibody to Calnexin, followed by Alexa-594-conjugated donkey anti-mouse IgG (red channel; A-D). Stained cells were analyzed by fluorescence microscopy. Merging of the images in the green and red channels generated the third image in each row; yellow indicates overlapping localization of the green and red channels. Arrows indicate puncta of colocalization. Bar, 10 μm.

Next, we examined by fluorescence microscopy the effect of IDN5706 on the intracellular localization of APP. We have previously shown that APP is mostly restricted to endosomes [10,20]. We observed a similar localization in cells stably expressing an shRNA to luciferase (Fig 8A). In contrast, treatment with IDN5706 revealed a dramatic change in the intracellular localization of APP, in a pattern that included distribution at the perinuclear region and at a cytoplasmic, reticular structure that resembled the ER (Fig 8B). This last assumption was confirmed by colocalization analysis of APP with the ER resident protein Calnexin [50] (Fig 8B; *r* = 0.946 ± 0.004). We found that 24.8 ± 6.0% of APP overlapped with Calnexin in response to IDN5706 treatment (S8 Fig, panel A), confirming that IDN5706 accumulates iAPP at the ER, and explaining the resulting iAPP distinct glycosylation. We next evaluated whether the accumulation of iAPP caused by IDN5706 could be lessened by autophagy. To test this possibility, we analyzed by fluorescence microscopy the accumulation of APP-GFP in response to IDN5706 in H4 cells stably expressing APP-GFP and stably depleted of Atg5. We observed that at steady state the amount and distribution of APP-GFP was not different in cells stably expressing Atg5 shRNA (Fig 8C), compared to cells stably expressing luciferase shRNA (Fig 8A). Quantification analysis confirmed this conclusion (S8 Fig, panel B). Cells depleted of Atg5 also showed redistribution of APP-GFP to the ER in response to IDN5706, demonstrated by colocalization analysis with Calnexin (*r* = 0.966 ± 0.012) (Fig 8D). This resulted in increased levels and increased accumulation of APP-GFP at the ER (Fig 8D). Quantification analysis also confirmed this conclusion, showing a significant 2.7 fold increase in the fluorescent signal of APP-GFP (S8 Fig, panel C), with 96.9 ± 2.9% of APP-GFP overlapping with Calnexin (S8 Fig, panel A). Unexpectedly, we also observed APP-GFP in remarkable structures scattered in the cytoplasm (Fig 8D). Based on the localization of Calnexin, these structures seemed to be part of the ER (Fig 8D). These results strongly suggest that IDN5706 triggers the entry of APP in ER-associated structures that favor the degradation of APP by Atg5-dependent autophagy.

**Fig 9:**
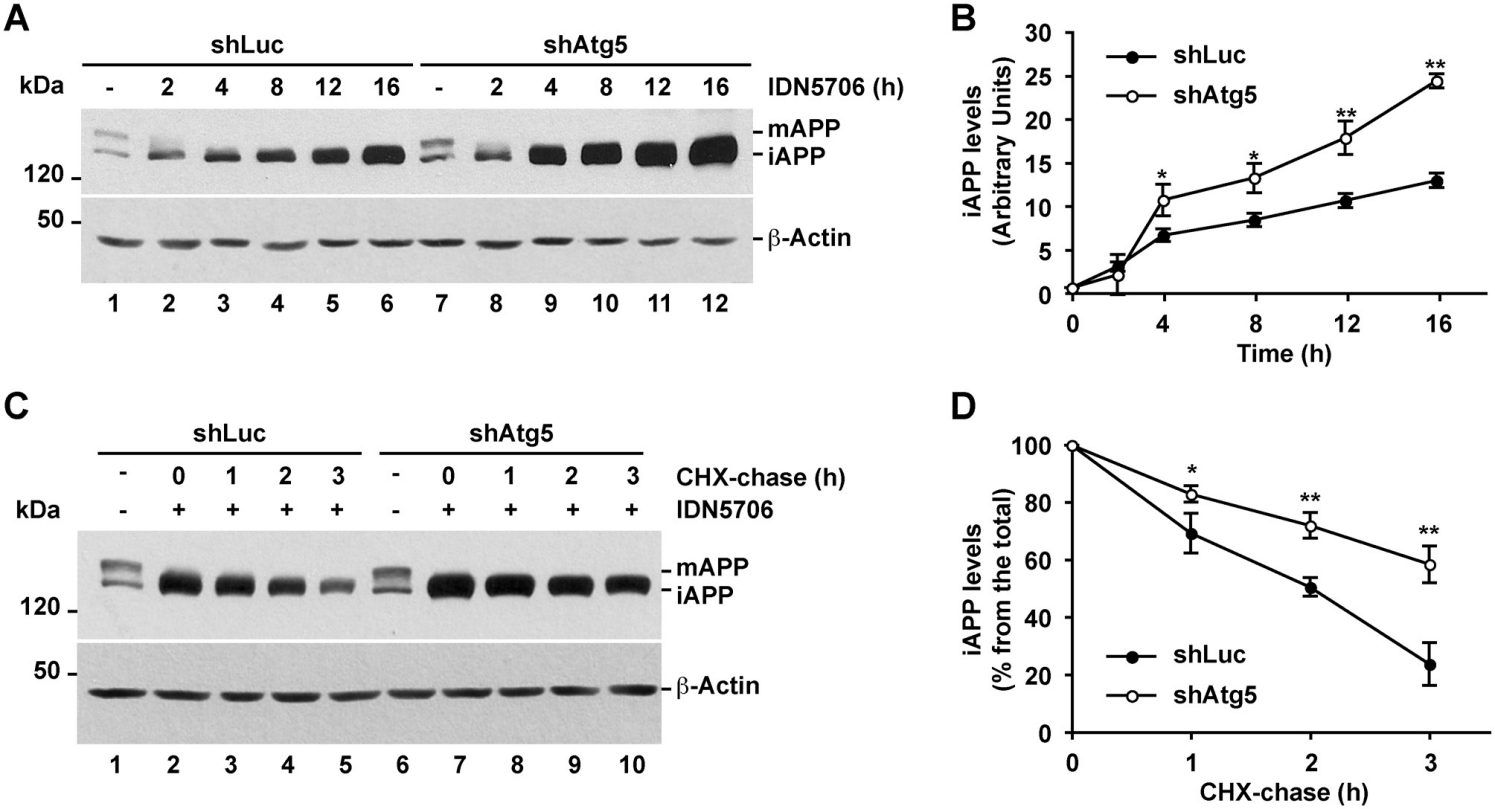
Accumulated immature APP in response to IDN5706 is degraded by Atg5-dependent autophagy. (A) H4 cells stably expressing an amyloidogenic version of APP tagged to GFP, and stably expressing either luciferase shRNA (control; shLuc) or Atg5 shRNA (shAtg5), were treated with 250 μM IDN5706 for the indicated periods of time, or (C) were left untreated (lanes 1 and 6) or treated with 250 μM IDN5706 (lanes 2–5 and lanes 7–10) for 12 h, followed by cycloheximide-chase with 150 μg/ml cycloheximide and 40 μg/ml chloramphenicol for 1–3 h in the presence of 250 μM IDN5706 (lanes 2–5 and lanes 7–10). Equivalent amounts of cell extracts were subjected to SDS-PAGE in 7.5% acrylamide gels, followed by Western blotting with an antibody to GFP. Western blotting with antibody to β-actin was used as loading control. mAPP, mature APP; iAPP, immature APP. The position of molecular mass markers is indicated on the left. (B and D) Densitometric quantification of the levels of iAPP shown either in A or C. The values depicted in the graphs represent the mean ± SD of three independent experiments. *, *P* < 0.05; **, *P* < 0.01.

We next evaluated by Western blot the levels of APP-GFP in cells stably expressing either luciferase shRNA or Atg5 shRNA. We observed that the levels of APP-GFP were not significantly different in cells depleted of Atg5 compared to control cells (Fig 9A, lanes 1 and 7; Fig 9C, lanes 1 and 6), indicating that APP is not significantly degraded by autophagy under basal conditions, in agreement with the analysis by fluorescent microscopy (Fig 8A and 8C). We observed a similar result when we analyzed the steady-state levels of endogenous APP in H4 cells stably depleted of Atg5 (data not shown). In contrast, cells depleted of Atg5 that were treated with IDN5706 showed significantly higher levels of iAPP-GFP (Fig 9A and 9B), suggesting that IDN5706 elicits the early degradation of iAPP-GFP via Atg5-dependent autophagy. To test this hypothesis, we analyzed the effect of IDN5706 in the turnover of iAPP-GFP in either control or Atg5-depleted H4 cells. Cells were pre-treated with IDN5706 for 16 h and, to prevent new protein synthesis, were further incubated for different periods of time with CHX and chloramphenicol (CHX-chase in the presence of IDN5706). Although IDN5706 treatment increased the levels of iAPP-GFP (Fig 9A), CHX-chase revealed that iAPP-GFP has a rapid turnover of ∼2 h (Fig 9C, lanes 1 to 5, and Fig 9D). In contrast, the turnover of iAPP-GFP in Atg5-depleted cells was significantly delayed to ∼3.5 h (Fig 9C, lanes 6 to 10), demonstrating that upon IDN5706 treatment the degradation of iAPP-GFP is facilitated by Atg5-dependent autophagy.

## Discussion

All proteins in eukaryotic cells undergo turnover, and different levels of synthesis and degradation are tightly regulated to maintain proper proteostasis [51]. Growing evidence shows that a wide variety of chemical compounds that seem to promote longevity and healthy aging are capable of modulating protein turnover pathways [52]. However, the elucidation of the mechanisms by which these compounds function is often elusive. In this study, we present evidence suggesting that IDN5706, a synthetic derivative of the chemical compound hyperforin of the St John's Wort plant, modulates EDEM1 levels, a key component of the ERAD pathway and, at the same time, enhances early degradation of iAPP via Atg5-dependent autophagy.

**Fig 10:**
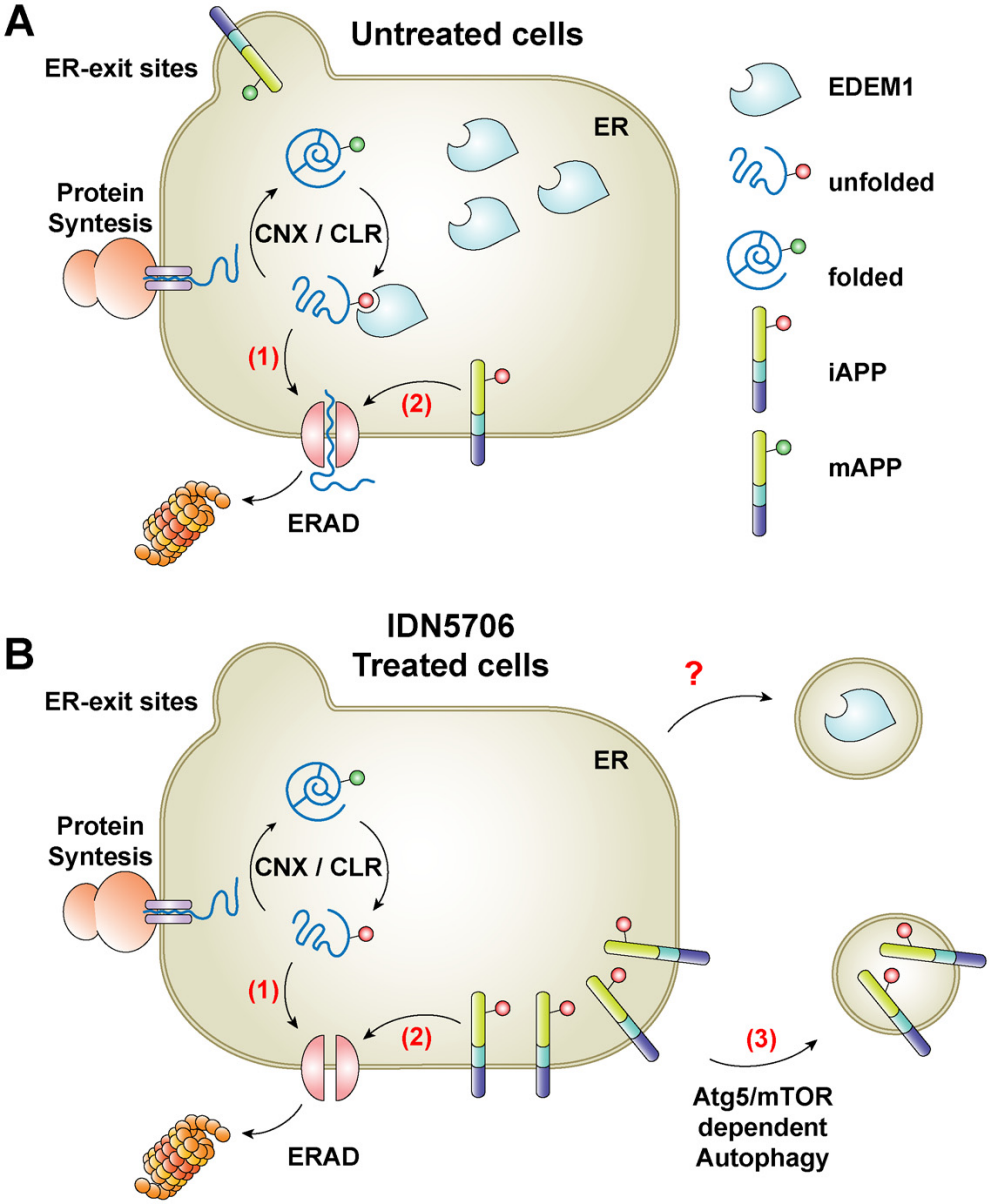
Effects of IDN5706 on APP turnover at the ER. (A) Similar to unfolded luminal glycoproteins that are recruited by EDEM1 from the Calnexin/Calreticulum (CNX/CLR) folding cycle (**1**), a fraction of newly-synthesized, immature APP (iAPP) is substrate of the endoplasmic reticulum-associated protein degradation (ERAD) (**2**). (B) The levels of EDEM1 are reduced upon IDN5706 treatment by a process independent on the levels of Atg5. This is a mechanism that needs further elucidation (**?**). Accumulation of iAPP in the ER elicited by IDN5706 activates an alternative early degradation of iAPP by Atg5-dependent autophagy (**3**).

ERAD functions by eliminating proteins that fail to adopt their native structure after translocation into the ER. However, this pathway is easily saturated, leading to the inappropriate accumulation of potentially toxic protein species that can lead to ER stress [53]. Moreover, increasing evidence shows that ERAD also controls the degradation of folded ER proteins [54], playing an important role in the regulation of the levels of APP and its C-terminal fragments [10,25], highlighting the function of ERAD in different processes. Importantly, the level of EDEM1 in the ER acts as a checkpoint for ERAD function [55], whereby knockdown or overexpression of EDEM1 results in inhibited or accelerated degradation of certain ERAD substrates [11,21,23,24]. Interestingly, ERAD inhibition has been shown to activate autophagy [56], a pathway that is considered to act as a second ERAD pathway, playing a crucial role as a survival mechanism. In this study, we show that IDN5706, which we have shown to prevent neuropathological changes in a mouse model of AD [6,7], reduces the protein levels of EDEM1 and activates autophagy (Fig 10). To investigate whether depletion of EDEM1 could be the trigger for activation of autophagy by IDN5706, we investigated the outcome of stable depletion of EDEM1. Contrary to a previous work with NSC34 cells [57], we observed that depletion of EDEM1 in H4 cells caused no changes in LC3 levels (S9 Fig, lane 3 compared to lane 1) or in LC3-decorated structures (data not shown). Moreover, we observed that IDN5706 increases the levels of LC3-II in cells stably expressing either luciferase shRNA or EDEM1 shRNA (S9 Fig, lane 4 compared to lane 2). We concluded that activation of autophagy by IDN5706 is not due to a reduction in EDEM1 levels, but probably dependent on the inactivation of mTOR activity (Fig 4). We propose that reduction in EDEM1 levels, as we observed in response to IDN5706 treatment, is a cellular response to privilege autophagy over ERAD to assure the degradation of some accumulated and immaturely glycosylated proteins. It will be interesting to explore if reduction in EDEM1 levels relies on mTOR status. To date, it has been proposed that the levels of EDEM1 are controlled at the ER by a process of selective autophagy (Fig 10A) [26,39]. Our results show that the reduction in EDEM1 levels by IDN5706 is not affected by depletion of Atg5 (Fig 5), contrary to the turnover of EDEM1 in basal conditions [26,39], suggesting the involvement of a novel turnover pathway for EDEM1 at the ER.

Several studies have shown that the abnormal accumulation of proteins in the ER can be alleviated by autophagy [29,31], a phenotype that can be triggered by the addition of Brefeldin A [58] or tunicamycin [59], compounds that perturb the transport of several proteins out of the ER. In the present study, we show that IDN5706 disrupts APP glycosylation (Fig 7B), and stimulates the degradation of iAPP through Atg5-dependent autophagy (Figs 8 and 9), with a resulting reduction in the processing of APP to CTFs, which could have beneficial effects to Alzheimer's disease. Our results imply that this process is activated in response to the accumulation of iAPP in the ER, acting as a compensatory mechanism to deliver iAPP to lysosomes by an alternative route, instead of delivery by the classical anterograde route [20]. Our previous findings showing that the treatment with IDN5706 precluded the γ-secretase cleavage of EGFP-tagged CTFβ suggested that IDN5706 could be acting as inhibitor of γ-secretase [7]. However, the results shown here of full-length APP (Fig 6B) suggest another possibility. We now propose that the decrease in γ-secretase cleavage of CTFβ was consequence of CTFβ incorporation into autophagosomes-like structures at the ER, similar to the effect of IDN5706 on iAPP. In this regard, several intriguing issues remain to be addressed: for example, how is iAPP incorporated into autophagosomes? How is iAPP recognized as a substrate of autophagy? Does this event occur exclusively at the ER? The fact that IDN5706 decreases the levels of EDEM1 and iAPP, with a different dependency on Atg5, suggests the existence of two different turnover pathways at the level of the ER. These pathways could operate differentially to eliminate either resident proteins such as EDEM1, or newly-synthesized, non-resident glycoproteins like APP (Fig 10B). These two pathways could be functioning to avoid ER stress and the associated damage [28–31].

Finally, our results demonstrate that IDN5706 is a novel modulator of autophagy. This feature might well explain some of the protective effects of IDN5706 observed in a mouse model of AD [7]. Due to recent findings implicating autophagy in tau clearance [60], it would be interesting to explore whether autophagy activation by IDN5706 could also reduce the levels of tau and its aggregate forms. Taken together, our results indicate that IDN5706 promotes APP clearance by stimulating its early degradation via Atg5-dependent autophagy. We propose that positive modulation of this process is an attractive niche for therapeutic intervention to reduce amyloidogenic processing of APP and Aβ production.

## Supporting Information

S1 Fig IDN5706 does not affect the mRNA levels of EDEM1. H4 cells were left untreated or treated with 125 μM or 250 μM IDN5706 for 16 h, and the mRNA levels of EDEM1 were analyzed by semiquantitative RT-PCR, and compared to the mRNA levels of GAPDH used as control.
(TIF)

S2 Fig Effect of tunicamycin on EDEM1 electrophoretic mobility. H4 cells were left untreated (lane 1 and 3) or treated either with 250 μM IDN5706 for 16 h (lane 2) or 2.5 μg/ml tunicamycin for 12 h (lane 4), followed by Western blotting with an antibody to EDEM1. The treatment with tunicamycin revealed unglycosylated, immature EDEM1 (iEDEM1). mEDEM1, mature EDEM1.
(TIF)

S3 Fig IDN5706 does not affect cell viability. H4 cells were left untreated or treated with 250 μM IDN5706 for 16 h, and cell viability was assessed by an MTT assay. Bars represent the mean ± SD of four independent experiments. NS, not significant.
(TIF)

S4 Fig IDN5706 increases the fluorescent signal of GFP-LC3 in NRK cells. Normal rat kidney (NRK) cells stably expressing GFP-LC3 were left untreated or treated with 250 μM IDN5706 for 16 h, and analyzed by fluorescence microscopy. Bars represent the mean ± SD of the fluorescent signal of GFP-LC3 in ten sets of images. ***, *P* < 0.001.
(TIF)

S5 Fig Turnover of LC3-II in response to IDN5706. H4 cells were left untreated (lanes 1–5) or treated with 250 μM IDN5706 (lanes 6–10) for 16 h, followed by cycloheximide-chase with 150 μg/ml cycloheximide and 40 μg/ml chloramphenicol (CHX-chase) for 1–4 h in the presence of 250 μM IDN5706. Cell extracts were subjected to Western blot analysis with an antibody to LC3. LC3-I, non-lipidated LC3; LC3-II, lipidated LC3. Western blotting with antibody to β-actin was used as loading control. The position of molecular mass markers is indicated on the left.
(TIF)

S6 Fig IDN5706 treatment does not perturb autophagic flux. H4 cells of human neuroglioma stably expressing mRFP-EGFP-LC3 were left untreated or treated with either EBSS for 2 h, 0.1 mM Chloroquine (CQ) for 2 h, or 250 μM IDN5706 for 8 h, and analyzed by fluorescence microscopy. Bars represent the mean ± SD of the mRFP-LC3-only fluorescent signal (mRFP^+^EGFP^-^) of ten sets of images. NS, not significant; ***, *P* < 0.001.
(TIF)

S7 Fig IDN5706 accumulates endogenous immature APP in a time-dependent manner. H4 cells were left untreated (lane 1) or treated with 250 μM IDN5706 for the indicated periods of time (lane 2–6). Cell extracts were subjected to Western blot analysis using the antibody anti-tail to the cytosolic C-terminal region of APP. mAPP, mature APP; iAPP, immature APP. Western blotting with antibody to β-actin was used as loading control. The position of molecular mass markers is indicated on the left.
(TIF)

S8 Fig Accumulation of APP-GFP at the ER by IDN5706 increases in cells depleted of Atg5. H4 cells stably expressing an amyloidogenic version of APP tagged to GFP, and stably expressing either luciferase shRNA (control; shLuc) or Atg5 shRNA (shAtg5), were treated with 250 μM IDN5706 for 8 h (A and C) or left untreated (B). Cells were fixed, and labeled with a mouse monoclonal antibody to Calnexin, followed by Alexa-594-conjugated donkey anti-mouse IgG (red channel; A-C). Stained cells were analyzed by fluorescence microscopy. Bars represent the mean ± SD of ten sets of images of APP-GFP indicating either overlapping between APP and Calnexin (A), or GFP-fluorescent signal (B and C). ***, *P* < 0.001; NS, not significant.
(TIF)

S9 Fig Depletion of EDEM1 does not affect the endogenous levels of LC3-II. H4 cells stably expressing a control luciferase shRNA (shLuc) or an EDEM1-specific shRNA (shEDEM1) were left untreated (lanes 1 and 3), or treated with 250 μM IDN5706 for 8 h (lanes 2 and 4). Cell extracts were subjected to Western blot analysis with specific antibodies to LC3 and EDEM1. LC3-I, non-lipidated LC3; LC3-II, lipidated LC3; mEDEM1, mature EDEM1. The asterisk indicates a band detected only in H4 cells. Western blotting with antibody to β-actin was used as loading control. The position of molecular mass markers is indicated on the left.
(TIF)
