# Supplementary figures and images for: Tetrahydrohyperforin Inhibits the Proteolytic Processing of Amyloid Precursor Protein and Enhances Its Degradation by Atg5-Dependent Autophagy
Source: PLoS One. 2015 Aug 26;10(8):e0136313. doi: 10.1371/journal.pone.0136313 (PMC4550396; doi:10.1371/journal.pone.0136313)

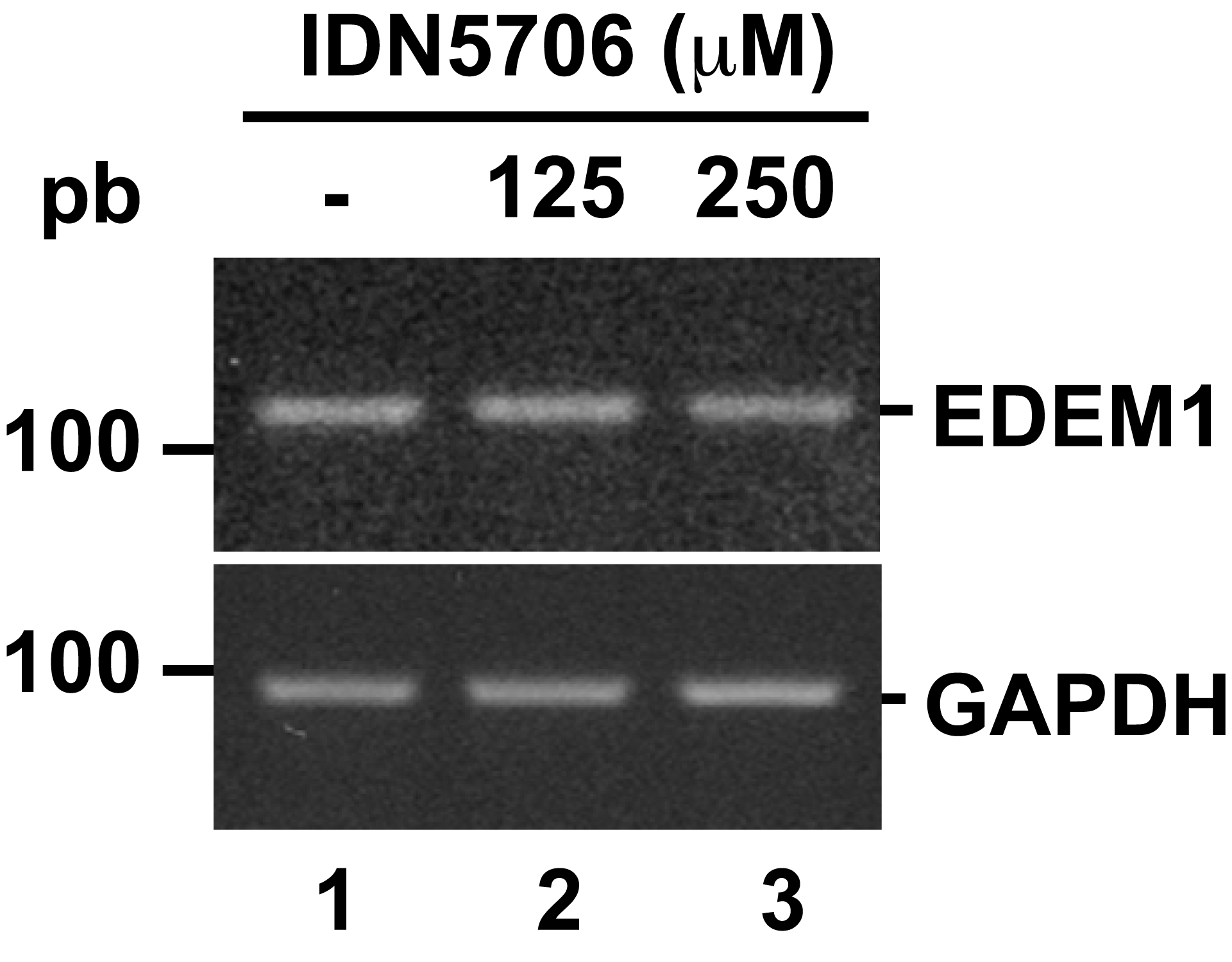

Supplement: S1 Fig — H4 cells were left untreated or treated with 125 μM or 250 μM IDN5706 for 16 h, and the mRNA levels of EDEM1 were analyzed by semiquantitative RT-PCR, and compared to the mRNA levels of GAPDH used as control. (TIF) [file pone.0136313.s001.tif]

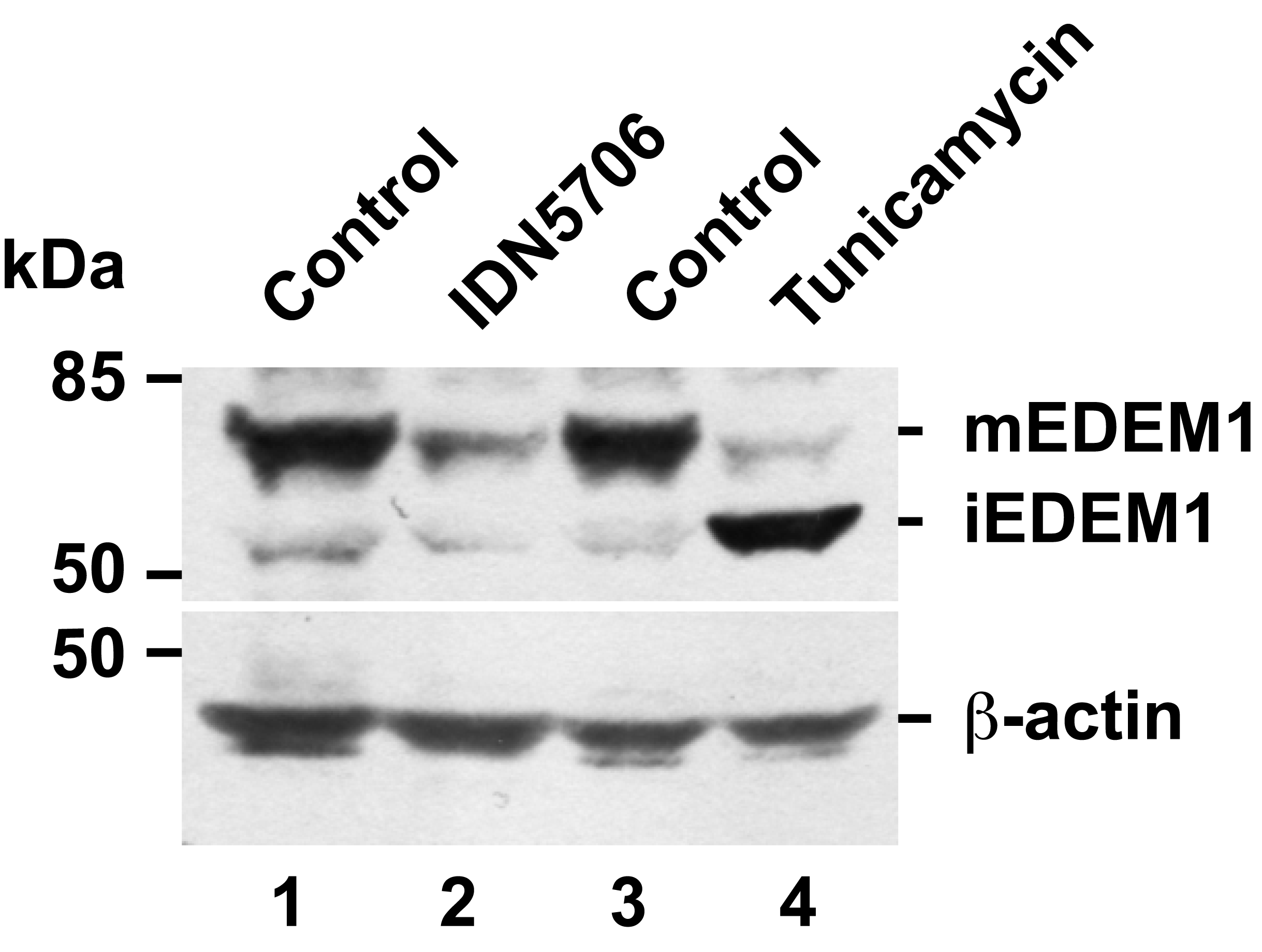

Supplement: S2 Fig — H4 cells were left untreated (lane 1 and 3) or treated either with 250 μM IDN5706 for 16 h (lane 2) or 2.5 μg/ml tunicamycin for 12 h (lane 4), followed by Western blotting with an antibody to EDEM1. The treatment with tunicamycin revealed unglycosylated, immature EDEM1 (iEDEM1). mEDEM1, mature EDEM1. (TIF) [file pone.0136313.s002.tif]

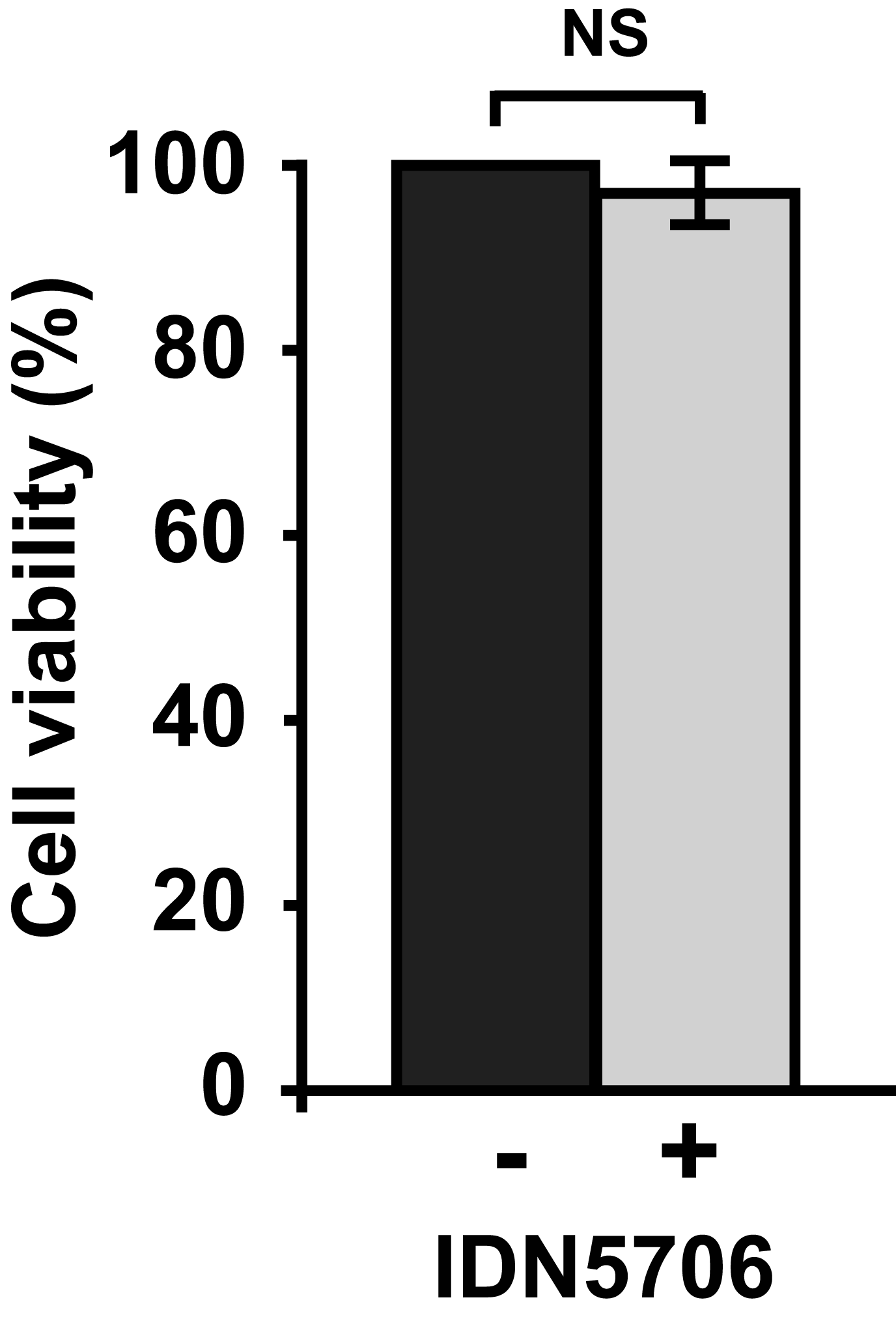

Supplement: S3 Fig — H4 cells were left untreated or treated with 250 μM IDN5706 for 16 h, and cell viability was assessed by an MTT assay. Bars represent the mean ± SD of four independent experiments. NS, not significant. (TIF) [file pone.0136313.s003.tif]

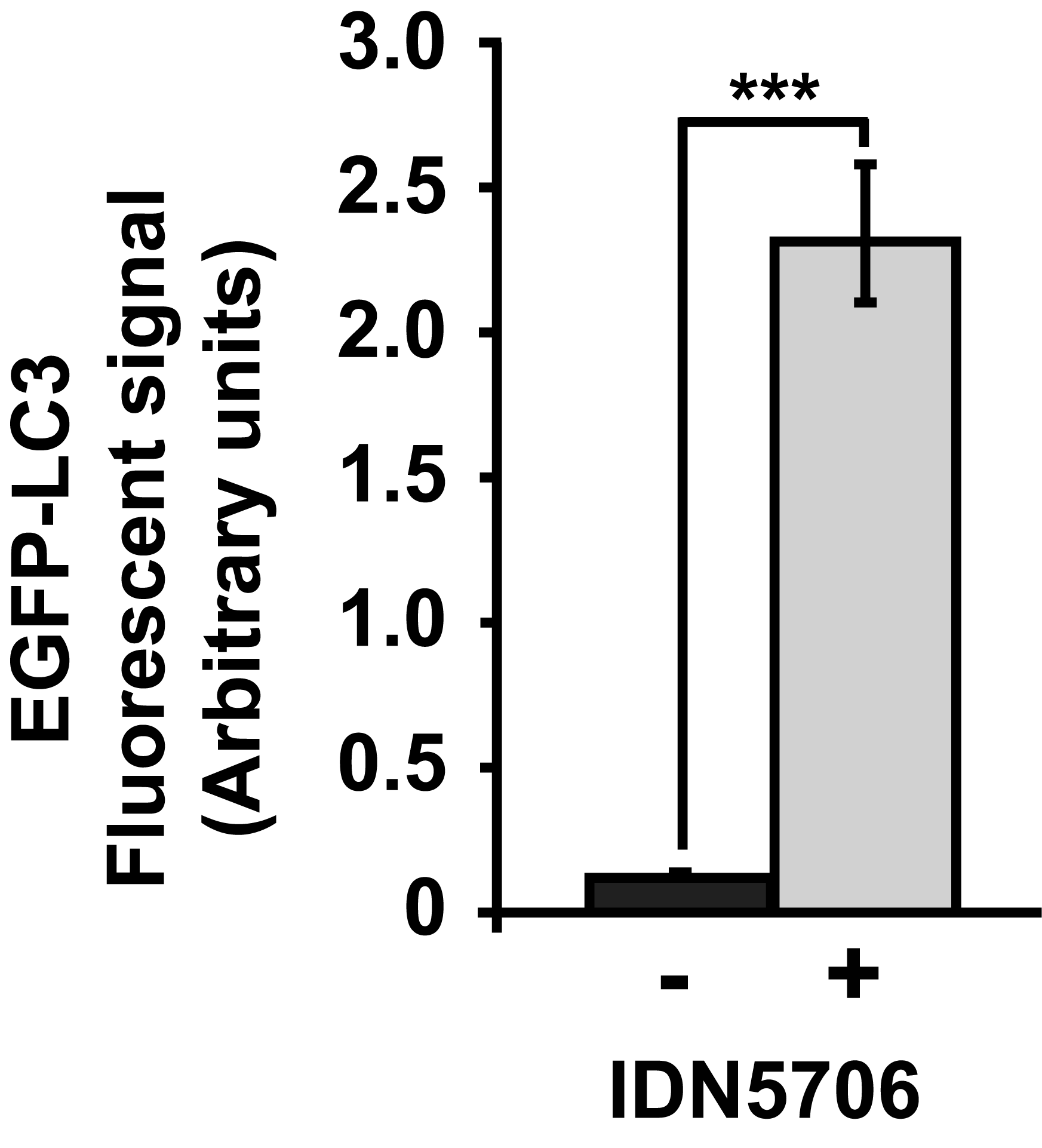

Supplement: S4 Fig — Normal rat kidney (NRK) cells stably expressing GFP-LC3 were left untreated or treated with 250 μM IDN5706 for 16 h, and analyzed by fluorescence microscopy. Bars represent the mean ± SD of the fluorescent signal of GFP-LC3 in ten sets of images. ***, P < 0.001. (TIF) [file pone.0136313.s004.tif]

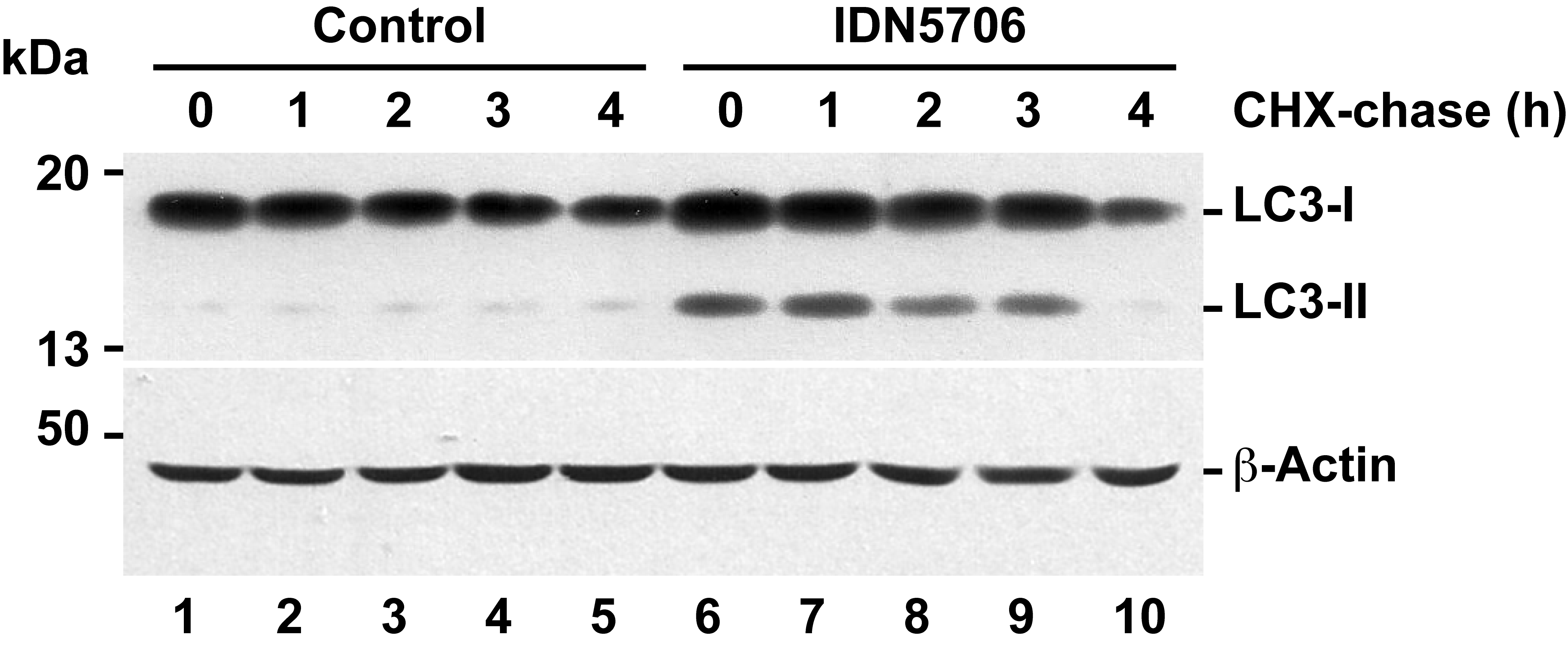

Supplement: S5 Fig — H4 cells were left untreated (lanes 1–5) or treated with 250 μM IDN5706 (lanes 6–10) for 16 h, followed by cycloheximide-chase with 150 μg/ml cycloheximide and 40 μg/ml chloramphenicol (CHX-chase) for 1–4 h in the presence of 250 μM IDN5706. Cell extracts were subjected to Western blot analysis with an antibody to LC3. LC3-I, non-lipidated LC3; LC3-II, lipidated LC3. Western blotting with antibody to β-actin was used as loading control. The position of molecular mass markers is indicated on the left. (TIF) [file pone.0136313.s005.tif]

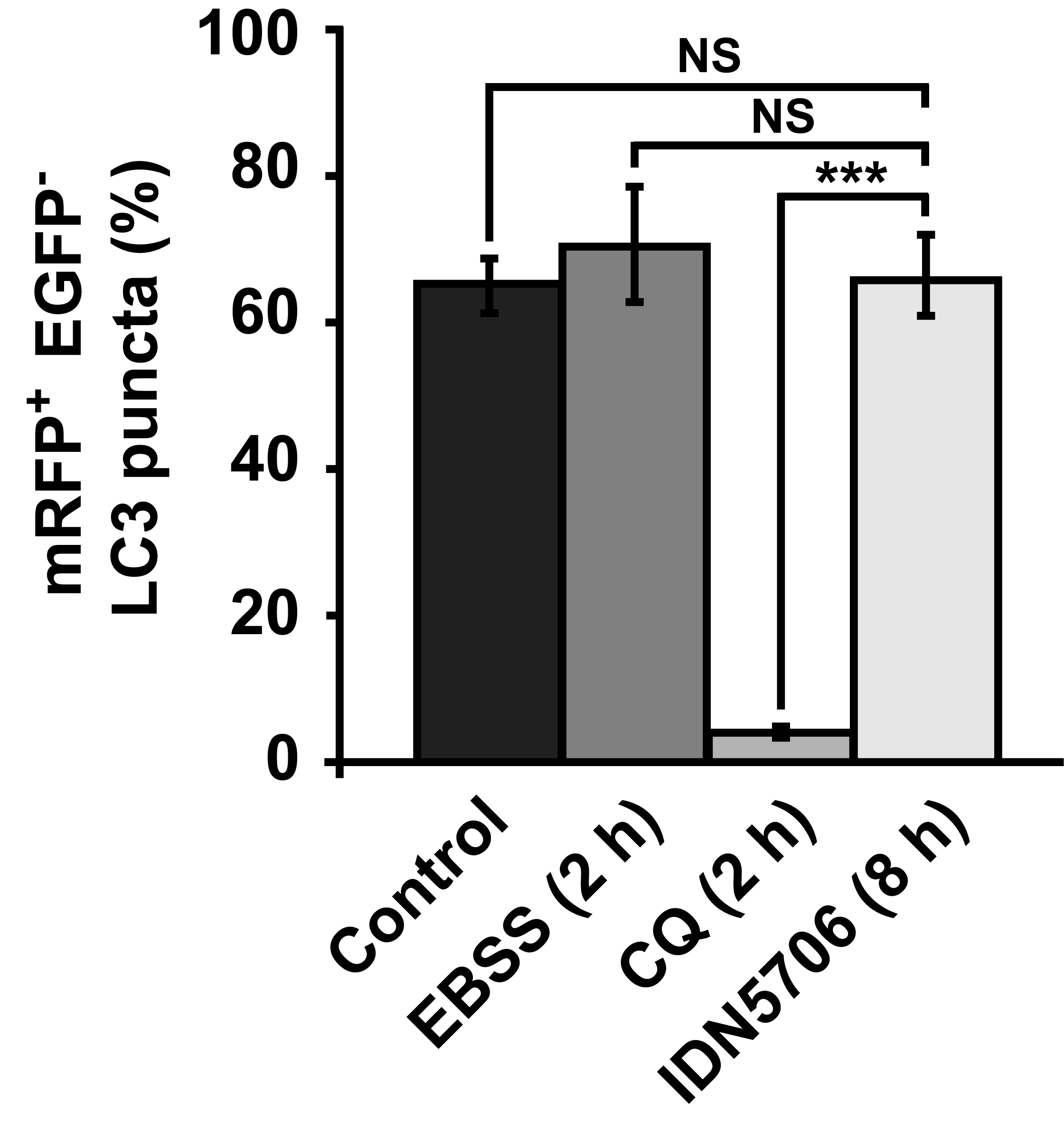

Supplement: S6 Fig — H4 cells of human neuroglioma stably expressing mRFP-EGFP-LC3 were left untreated or treated with either EBSS for 2 h, 0.1 mM Chloroquine (CQ) for 2 h, or 250 μM IDN5706 for 8 h, and analyzed by fluorescence microscopy. Bars represent the mean ± SD of the mRFP-LC3-only fluorescent signal (mRFP+EGFP-) of ten sets of images. NS, not significant; ***, P < 0.001. (TIF) [file pone.0136313.s006.tif]

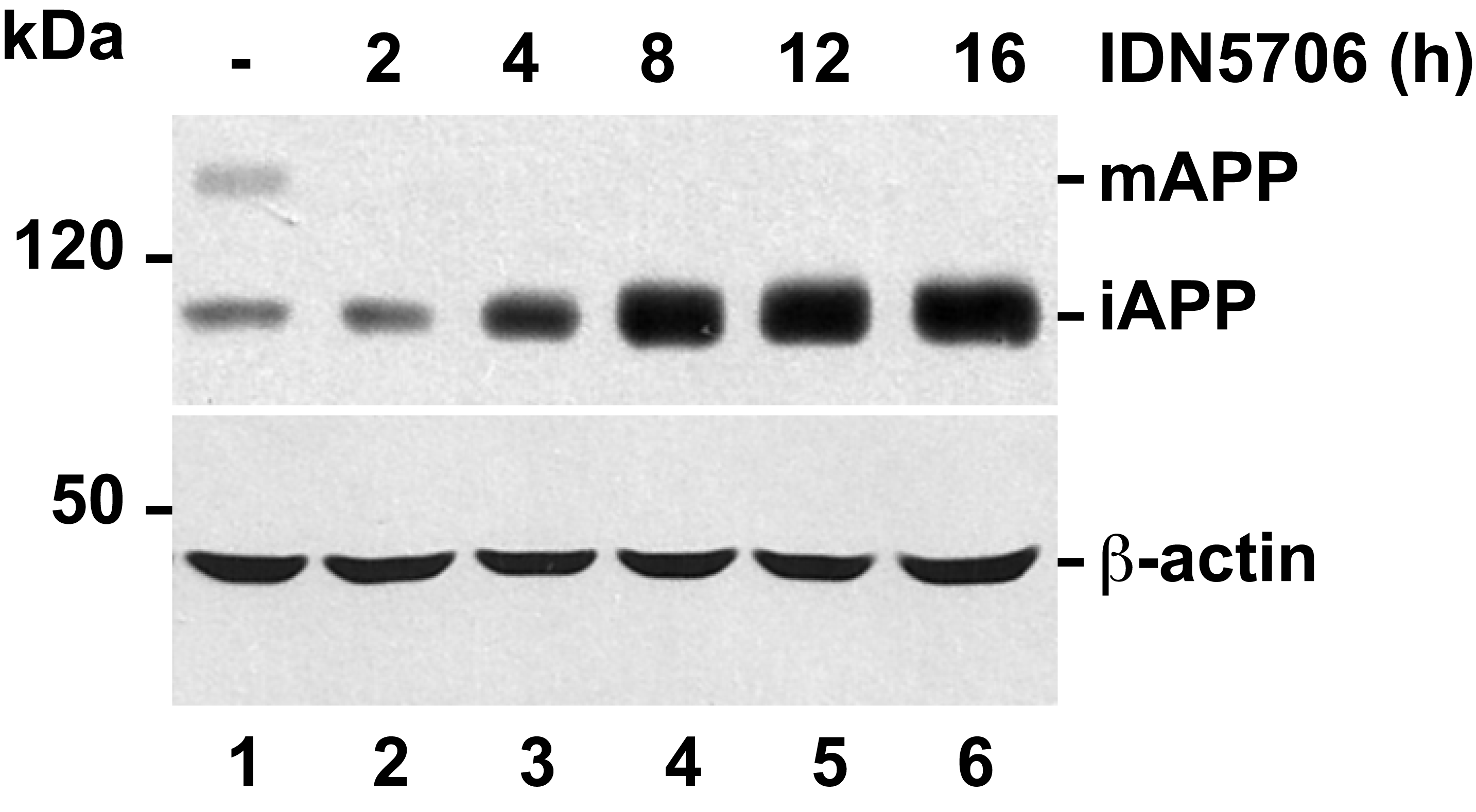

Supplement: S7 Fig — H4 cells were left untreated (lane 1) or treated with 250 μM IDN5706 for the indicated periods of time (lane 2–6). Cell extracts were subjected to Western blot analysis using the antibody anti-tail to the cytosolic C-terminal region of APP. mAPP, mature APP; iAPP, immature APP. Western blotting with antibody to β-actin was used as loading control. The position of molecular mass markers is indicated on the left. (TIF) [file pone.0136313.s007.tif]

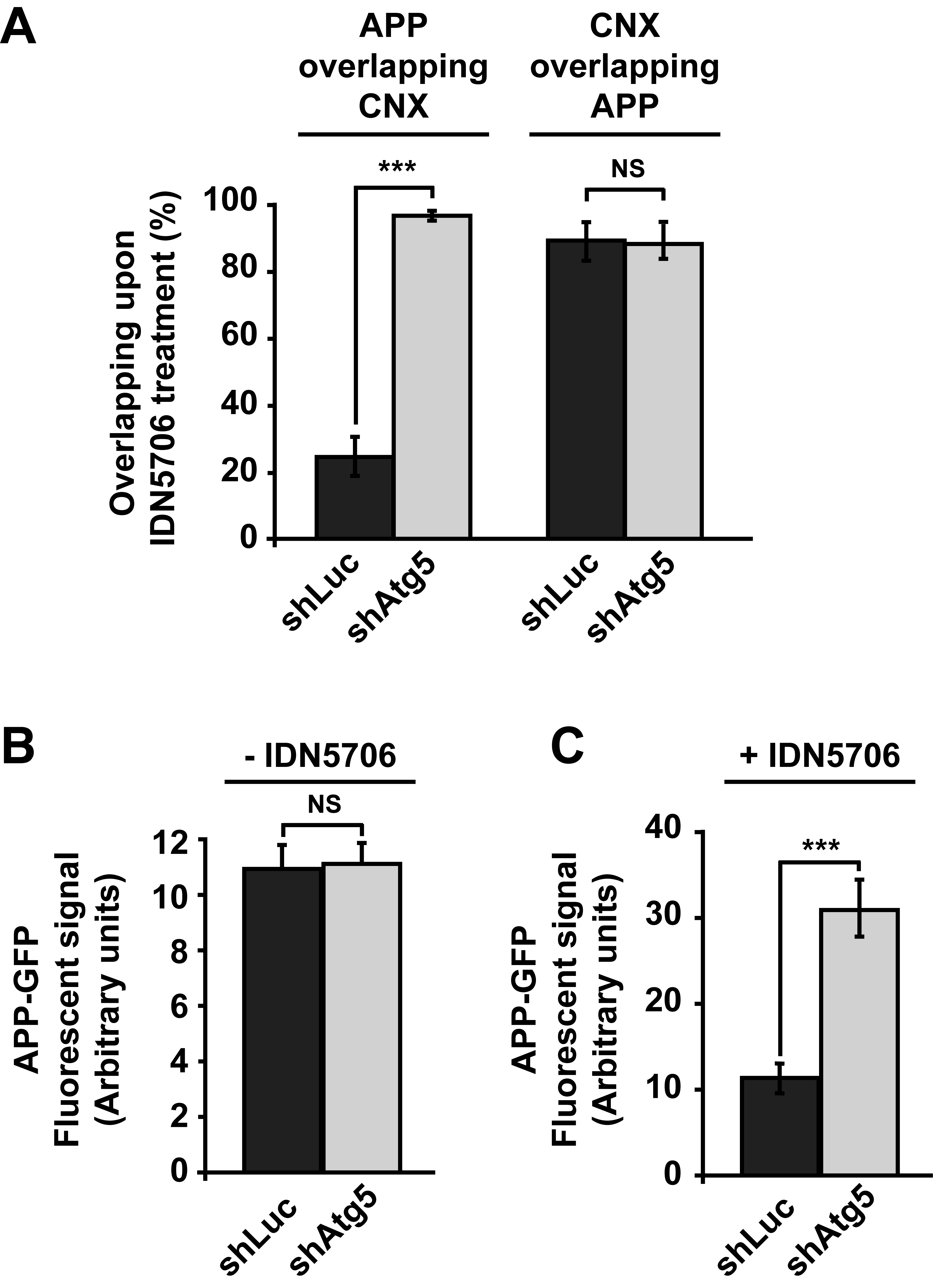

Supplement: S8 Fig — H4 cells stably expressing an amyloidogenic version of APP tagged to GFP, and stably expressing either luciferase shRNA (control; shLuc) or Atg5 shRNA (shAtg5), were treated with 250 μM IDN5706 for 8 h (A and C) or left untreated (B). Cells were fixed, and labeled with a mouse monoclonal antibody to Calnexin, followed by Alexa-594-conjugated donkey anti-mouse IgG (red channel; A-C). Stained cells were analyzed by fluorescence microscopy. Bars represent the mean ± SD of ten sets of images of APP-GFP indicating either overlapping between APP and Calnexin (A), or GFP-fluorescent signal (B and C). ***, P < 0.001; NS, not significant. (TIF) [file pone.0136313.s008.tif]

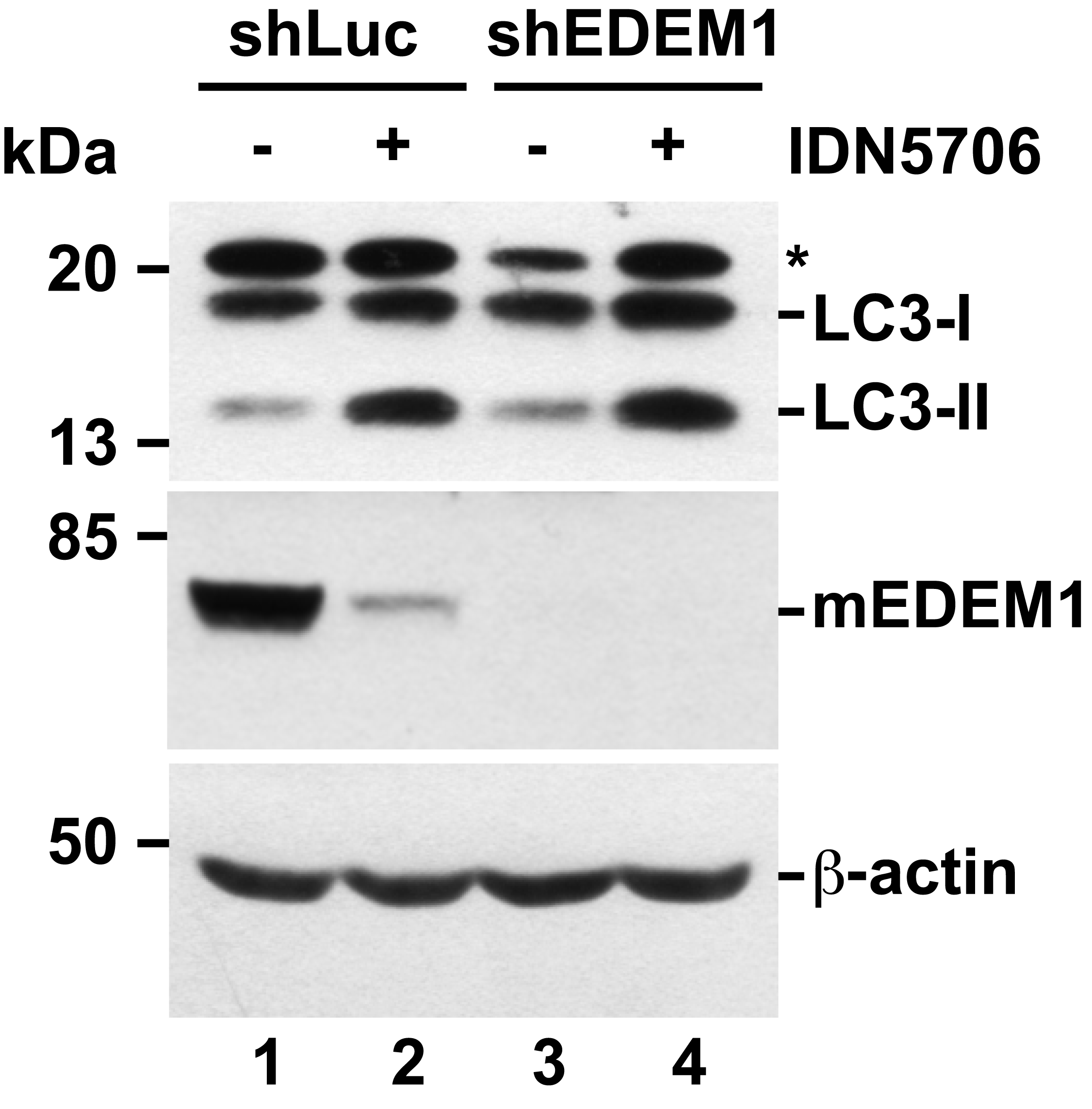

Supplement: S9 Fig — H4 cells stably expressing a control luciferase shRNA (shLuc) or an EDEM1-specific shRNA (shEDEM1) were left untreated (lanes 1 and 3), or treated with 250 μM IDN5706 for 8 h (lanes 2 and 4). Cell extracts were subjected to Western blot analysis with specific antibodies to LC3 and EDEM1. LC3-I, non-lipidated LC3; LC3-II, lipidated LC3; mEDEM1, mature EDEM1. The asterisk indicates a band detected only in H4 cells. Western blotting with antibody to β-actin was used as loading control. The position of molecular mass markers is indicated on the left. (TIF) [file pone.0136313.s009.tif]
